# Fatty acid oxidation facilitates DNA double-strand break repair by promoting PARP1 acetylation

**DOI:** 10.1038/s41419-023-05968-w

**Published:** 2023-07-15

**Authors:** Seungyeon Yang, Sunsook Hwang, Byungjoo Kim, Seungmin Shin, Minjoong Kim, Seung Min Jeong

**Affiliations:** Department of Biochemistry, Institute for Aging and Metabolic Diseases, Department of Biomedicine & Health Sciences, College of Medicine, The Catholic University of Korea, 222, Banpo-daero, Seocho-gu, Seoul, 06591 South Korea

**Keywords:** DNA, Fatty acids, Homologous recombination

## Abstract

DNA repair is a tightly coordinated stress response to DNA damage, which is critical for preserving genome integrity. Accruing evidence suggests that metabolic pathways have been correlated with cellular response to DNA damage. Here, we show that fatty acid oxidation (FAO) is a crucial regulator of DNA double-strand break repair, particularly homologous recombination repair. Mechanistically, FAO contributes to DNA repair by activating poly(ADP-ribose) polymerase 1 (PARP1), an enzyme that detects DNA breaks and promotes DNA repair pathway. Upon DNA damage, FAO facilitates PARP1 acetylation by providing acetyl-CoA, which is required for proper PARP1 activity. Indeed, cells reconstituted with PARP1 acetylation mutants display impaired DNA repair and enhanced sensitivity to DNA damage. Consequently, FAO inhibition reduces PARP1 activity, leading to increased genomic instability and decreased cell viability upon DNA damage. Finally, our data indicate that FAO serves as an important participant of cellular response to DNA damage, supporting DNA repair and genome stability.

## Introduction

Cells encounter various types of DNA damage. Upon these threats, cells promote a well-coordinated signaling response known as the DNA damage response (DDR) [1, 2]. DNA repair is one of essential components of the DDR. When DNA double-strand breaks (DSBs) occur, cells initiate assembly of DNA damage repair machineries, such as homologous recombination (HR) or non-homologous end-joining (NHEJ), to repair these [3, 4]. Defects in DNA repair pathways lead to incorporation of mutations into genome and accumulation of chromosomal instability [3, 5, 6]. The importance of DNA repair is further highlighted by the fact that increased tumor incidence and/or accelerated aging phenotypes in patients with genetic disorders in DNA repair pathways [7, 8].

Although DNA repair pathways have been intensively investigated, little is known about the involvement of metabolism in DNA repair. Recently, an emerging body of evidence suggests that metabolic pathways can play crucial roles in DNA repair. For example, the pentose phosphate pathway is enhanced after DNA damage and contributes to the synthesis of nucleotide precursors for DNA repair [9]. Additionally, it has been shown that repression of glutamine anaplerosis is necessary for proper cell cycle arrest and DNA repair upon genotoxic stress [10]. In turn, identification of new metabolic regulators of DNA repair could provide important insights into the cellular metabolic response to DNA damage.

Fatty acids are one of major fuels for cells and organisms. During periods of increased metabolic demand, fatty acids are broken down by a multistep process known as fatty acid oxidation (FAO) to produce FADH_2_, NADH and acetyl-CoA [11, 12]. FADH_2_ and NADH are used in the electron-transport chain to produce ATP through mitochondrial oxidative phosphorylation (OXPHOS) and acetyl-CoAs enter the tricarboxylic acid (TCA) cycle to refill the mitochondrial carbon pool [12]. As the DDR is an energetically expensive mechanism for cells to initiate cell cycle arrest and to activate DNA repair pathways, and thus massive DNA damage often leads to depletion of cellular energy [13–15], it has been proposed that induction of catabolic pathways might be required for the DDR. In support of this notion, a recent study has shown that genotoxic stress promotes FAO and OXPHOS to compensate a decline in cellular ATP levels [15], illustrating that the induction of FAO could function as a metabolic adaptive response to DNA damage.

On the other hand, to rapidly respond to DNA damage, many proteins involved in the DDR are regulated by posttranslational modifications such as phosphorylation and acetylation [16, 17]. For example, acetylation of poly(ADP-ribose) polymerase 1 (PARP1) contributes to synthesis of poly(ADP-ribose) (PAR) polymers upon DNA damage and recruitment of DNA repair proteins to DNA lesions [18]. Because levels of cellular acetyl-CoA are indispensable determinant of acetylation [19, 20], and FAO is an important source of acetyl-CoA [21], it is not surprising that FAO has emerged as an important regulator of protein acetylation. Indeed, recent studies have found that acetyl-CoAs derived from FAO function as a major carbon source for acetylation of nuclear proteins as well as mitochondrial proteins [22, 23]. Thus, considering the pivotal role of FAO in acetyl-carbon supplement, FAO could serve as an important regulator of acetylation upon DNA damage. However, no study has yet investigated whether FAO contributes to the DDR by regulating protein acetylation rather than by supplying ATP.

Here, we described a novel function of FAO in the regulation of DNA repair. Using pharmacological or genetic perturbation of FAO, we demonstrated that FAO is crucial for HR repair. Moreover, we found that inhibition of FAO impedes PARP1 acetylation in response to DNA damage, which leads to impaired PARP1 activation and suppression of DNA repair. As a consequence, FAO inhibition results in increased genomic instability after genotoxic stress. Collectively, our work identifies FAO as an important player in the DDR that contributes to facilitating DNA repair and maintaining genome integrity.

## Results

### FAO is required for DSBs DNA repair

**Fig. 1:**
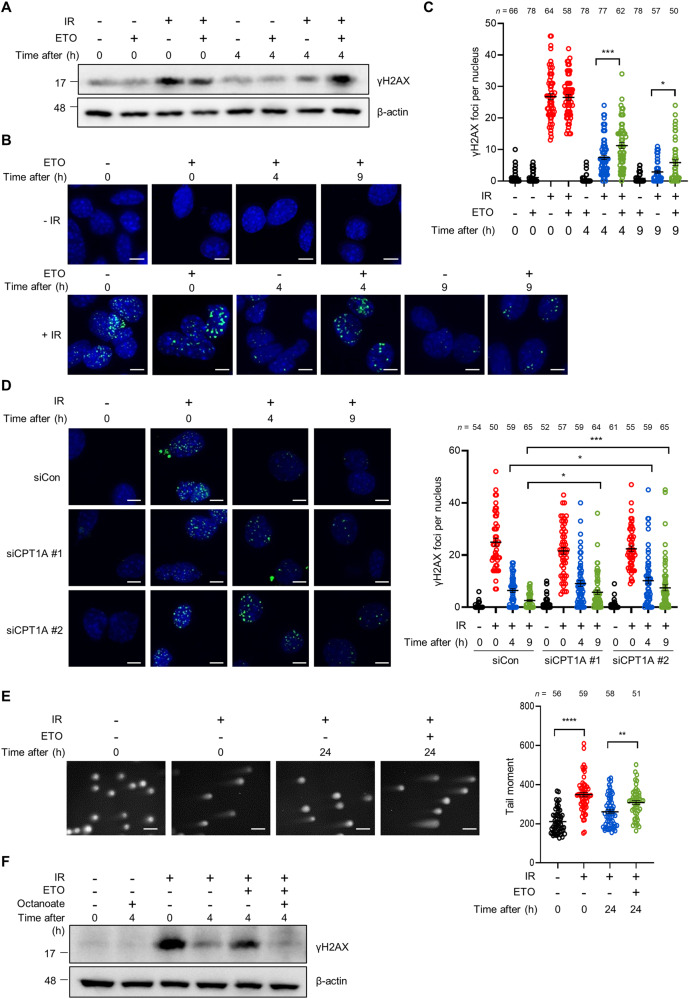
FAO supports DNA double-strand break repair. **A** γH2AX protein levels in immortalized MEFs exposed to 3 Gy IR and treated with or without 200 μM ETO for the indicated times. β-actin was used as a loading control. **B** Immortalized MEFs were exposed to 3 Gy IR and then treated with or without ETO for the indicated times. Immunofluorescent staining was performed with a nuclear marker (DAPI) and anti-γH2AX (green) on the indicated cells. Scale bar represents 6 μm. **C** Number of γH2AX foci per nucleus in the indicated cells as shown in (**B**). The number of cells analyzed per condition (n) is indicated. Statistical analysis performed using one-way ANOVA with Tukey’s multiple comparisons test. **D** Immortalized MEFs were transfected with siControl or two independent siRNAs against CPT1A and were exposed to 3 Gy IR. Representative images of immunofluorescence staining (γH2AX, Green; DAPI, Blue). Scale bar represents 6 μm. Number of γH2AX foci per nucleus as indicated (right). The number of cells analyzed is noted. Statistical analysis was performed using two-way ANOVA with Tukey’s multiple comparisons test. **E** Immortalized MEFs were exposed to 3 Gy IR and then recovered for 24 h with or without ETO. DNA-damaged cells were measured using a neutral comet assay. Tail moment values are shown (right). The number of cells analyzed per condition (n) is indicated. Statistical analysis was performed using one-way ANOVA with Tukey’s multiple comparisons test. Scale bar represents 2 mm. **F** Immortalized MEFs were exposed to 3 Gy IR and then treated with or without 200 μM ETO and/or 1 mM octanoate for the indicated times. Lysates were immunoblotted with anti-γH2AX antibody. **C**–**E** The number of cells was pooled from three independent experiments. All error bars ± SEM. *p < 0.05, **p < 0.01, ***p < 0.001 and ****p < 0.0001.

The induction of FAO pathway has recently been linked to cellular response to DNA damage [15], yet it remains to be resolved whether FAO could serve as a regulator of DNA repair. To assess the role of FAO in DNA repair, mouse embryonic fibroblasts (MEFs) were exposed to ionizing radiation (IR) and the levels of phosphorylation of the histone variant H2AX at Ser 139 (termed γH2AX), a marker of DSBs, were determined after FAO inhibition. IR induced γH2AX expression in cells, which was markedly decreased at 4 hours after IR exposure as DNA repair had been progressed (Fig. 1A). Notably, when cells were treated with etomoxir (ETO), which specifically impairs the import of fatty acids into mitochondria by inhibiting carnitine palmitoyl-transferase 1A (CPT1A), the restoration of increased γH2AX expression was significantly abrogated (Fig. 1A). Moreover, in a γH2AX clearance assay, ETO treated cells exhibited a decreased DNA repair capacity compared with control cells after IR exposure (Fig. 1B, C). Comparable results were observed in the presence of etoposide (ETS), a topoisomerase 2 inhibitor that causes DSBs [24] (Supplementary Fig. S1A, B). Consistent with these results, MEFs, in which CPT1A activity was inhibited by using short interfering RNAs (siRNAs) against CPT1A (Supplementary Fig. S1C), exhibited a delayed clearance of γH2AX foci after IR exposure (Fig. 1D). We also observed that knockdown of CPT1A impaired the restoration of γH2AX expression after IR exposure or ETS treatment (Supplementary Fig. S1D, E). To further confirm, we measured DNA strand breaks by performing comet assay. In line with our results, FAO inhibited cells exhibited higher tail moments than control cells after DNA damage (Fig. 1E). Lastly, to directly assess the role of FAO in mediating these effects, we subsequently treated cells with octanoate, a medium chain fatty acid, which can diffuse across mitochondrial membranes without the help of CPT1A and incorporate into the FAO spiral [22]. Indeed, octanoate treatment almost completely rescued the delayed restoration of γH2AX expression by CPT1A inhibition (Fig. 1F). Together, these data indicate that FAO may contribute to cellular response to genotoxic stress by supporting DNA repair.

### FAO contributes to HR repair

**Fig. 2:**
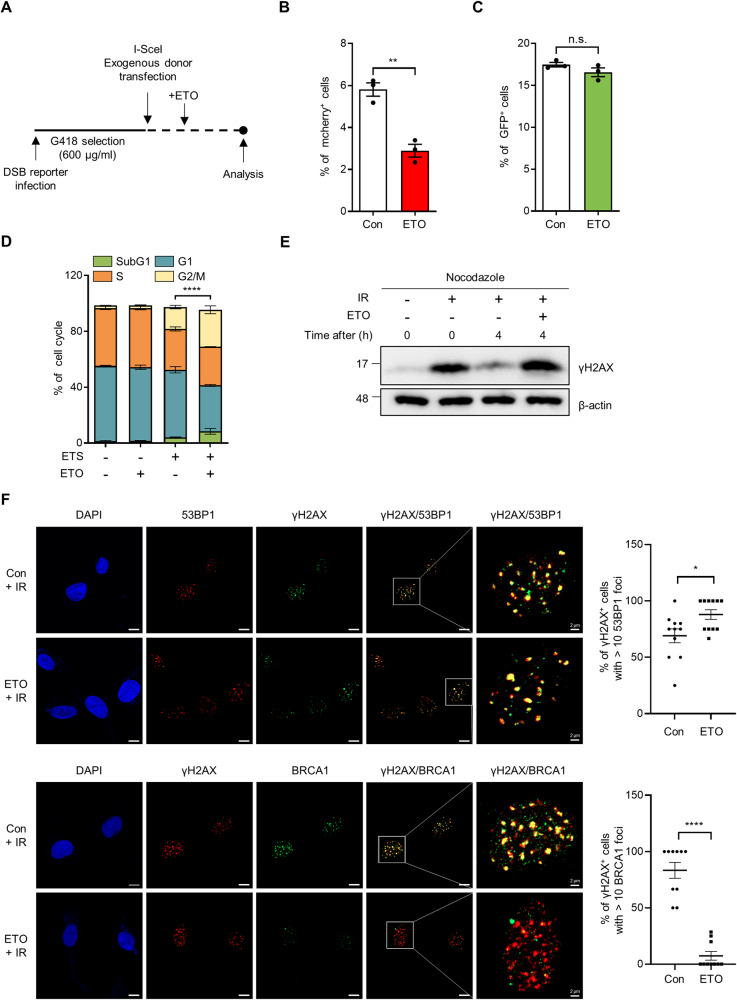
FAO affects HR repair by promoting BRCA1 recruitment. **A** Experimental design for Fig. 2B and C. 293 cells were infected with DSB reporter and selected with G418. Selected cells were transfected with exogenous donor and I-SceI. After transfection, cells were treated with or without ETO for 48 h and analyzed using FACS. Quantification of (**B**) mCherry and (**C**) GFP expression in BFP positive cells. Three million cells per sample were analyzed. Statistical analysis was based on two-tailed Student’s t-test. **D** Immortalized MEFs were treated with 0.5 μM ETS, 200 μM ETO, or both as indicated. The Cell cycle was analyzed by BrdU staining. Statistical analysis was performed using two-way ANOVA with Tukey’s multiple comparisons test. The indicated p-values (****) represent the comparative values between ETS-treated and ETS plus ETO-treated cells in G2/M phase. **E** γH2AX protein levels in immortalized MEFs. Cells, pre-treated with nocodazole (200 nM) for 20 h, were exposed to 3 Gy IR and then treated with or without 200 μM ETO for the indicated times. β-actin was used as loading control. **F** HeLa cells were exposed to 3 Gy IR and treated with or without ETO for 4 h. Representative images of immunofluorescence staining (53BP1, Red; γH2AX, Green; DAPI, Blue in upper panels and BRCA1, Green; γH2AX, Red; DAPI, Blue in lower panels). Scale bar represents 10 μm. Percentages of γH2AX positive cells with >10 Foci of BRCA1 or 53BP1 as indicated (right). Statistical analysis was performed using two-tailed Student’s t-test. All error bars ± SEM. *p < 0.05, **p < 0.01, and ****p < 0.0001.

DSBs in DNA are repaired by various pathways and there are two major mechanisms of DSB repair: HR or NHEJ [3, 4]. To assess how FAO supports DNA repair, we used fluorescence-based reporter system that allows the quantitative comparison of NHEJ and HR in the same cells [25], and the choice between these was analyzed (Fig. 2A). We found that FAO inhibition significantly impaired HR, whereas NHEJ was not affected in the same cells (Fig. 2B, C). The determination between HR and NHEJ is also regulated in a cell-cycle dependent manner [26]. While NHEJ is used throughout the cell cycle, HR is preferentially working during S and G2 phases. Because we found that FAO is required for HR, we examined the effects of FAO inhibition in cell cycle upon DNA damage. As previously reported [27], ETS treatment accumulated cells in G2/M phase (Fig. 2D). Importantly, when combined with FAO inhibition, this effect was significantly augmented (Fig. 2D), indicating that the reduced HR by FAO inhibition might further deteriorate the capability of cells escaping the G2/M arrest. Additionally, to exclude the possibility that the altered cell cycle arrest by FAO inhibition may cause the decreased DSB repair, we arrested cells at the G2/M phase by using nocodazole, which inhibits microtubule polymerization (Supplementary Fig. S2A), and then examined γH2AX expression after IR exposure. Importantly, we found that FAO inhibition impaired the restoration of increased γH2AX expression, even in the presence of nocodazole (Fig. 2E), indicating that the decreased DSB repair by FAO inhibition is not caused by the altered cell cycle arrest.

Breast cancer early onset 1 (BRCA1) and p53-binding protein 1 (53BP1) are known as key upstream factors for HR or NHEJ, respectively [28, 29]. To further confirm the importance of FAO in HR repair, we examined whether FAO affects the recruitment of these DNA repair-related proteins to DNA DSBs region. Whereas FAO inhibition had no effect on the number of 53BP1 foci after IR, the recruitment of BRCA1 was markedly inhibited in ETO-treated cells (Fig. 2F and Supplementary Fig. S2B). In addition, FAO inhibition significantly reduced colocalization of γH2AX with BRCA1 but not with 53BP1 (Fig. 2F). We also obtained similar results in CPT1A knockdown cells (Supplementary Fig. S2C, D). Together, these findings demonstrate that FAO is required for HR repair upon DNA damage.

### FAO potentiates PARP1 activity in response to DNA damage

**Fig. 3:**
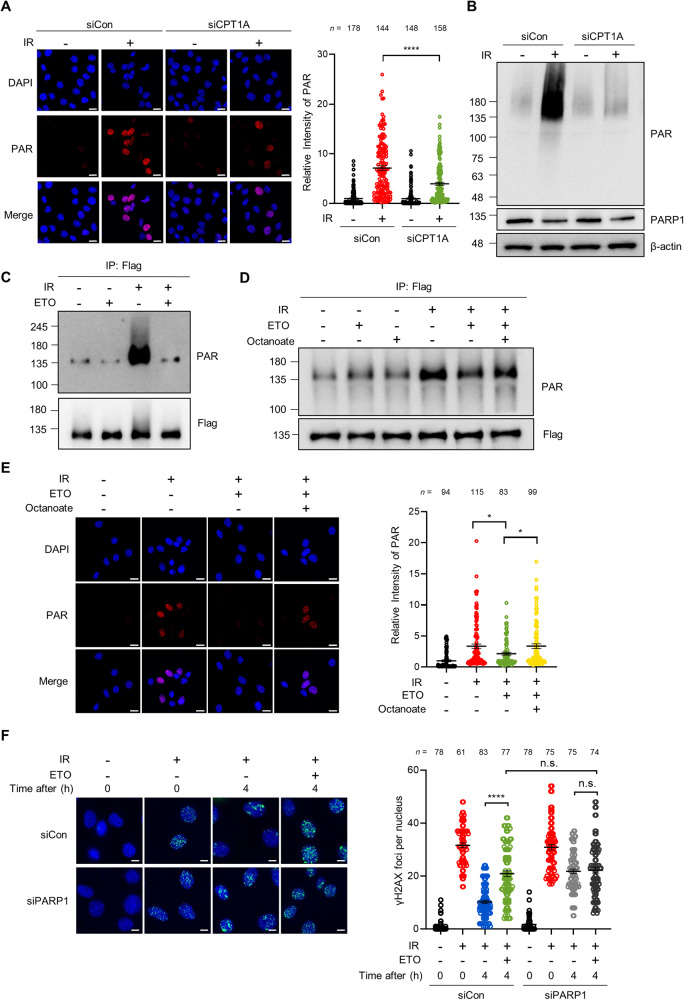
FAO participates in DNA repair by regulating PARP1 activity. **A** Immortalized MEFs, transfected with siControl or siRNAs against CPT1A, were pre-treated with 10 μM PDD00017273 for 1 h and then exposed to 3 Gy IR. Immunofluorescent staining was performed with anti-PAR antibody and DAPI. Scale bar represents 20 μm. Relative fluorescence intensity of PAR as indicated (right). Statistical analysis was based on two-way ANOVA with Tukey’s multiple comparisons test. The number of cells analyzed per condition (n) is indicated. **B** 293 T cells were transfected with siControl or siRNA against CPT1A and were treated with 10 μM PDD00017273 for 1 h before irradiation (10 Gy). Lysates were subjected to immunoblotting analysis with anti-PAR and anti-PARP1 antibodies. **C** 293 T cells were transfected with pCMV-PARP1-3x Flag. After transfection, cells were treated with or without ETO for 4 h and then irradiated 10 Gy IR. Lysates were immunoprecipitated with Flag antibody, followed by immunoblotting with anti-PAR antibody. **D** 293 T cells were treated with or without ETO and/or 1 mM octanoate for 4 h and then incubated with 10 μM PDD00017273 for 1 h before 10 Gy IR. Lysates were immunoblotted with anti-PAR antibody. **E** Immortalized MEFs were treated with or without ETO and/or 1 mM octanoate for 4 h and then incubated with 10 μM PDD00017273 for 1 h before 3 Gy IR. Immunofluorescent staining was performed with a nuclear marker (DAPI) and anti-PAR antibody on the indicated cells. Scale bar represents 20 μm. Relative fluorescence intensity of PAR as indicated (right). Statistical analysis was performed using one-way ANOVA with Tukey’s multiple comparisons test. The number of cells analyzed per condition (n) is noted. **F** Representative images of γH2AX foci per nucleus in Control or PARP1 knockdown MEF cells. Scale bar represents 6 μm. Number of γH2AX foci per nucleus as indicated (right). Statistical analysis was based on two-way ANOVA with Tukey’s multiple comparisons test. The number of cells analyzed per condition (n) is indicated. In (A), (E), and (F), the number of cells analyzed per condition was pooled from three independent experiments. All error bars ± SEM. *p < 0.05 and ****p < 0.0001.

PARP1 is one of the first signaling protein recruited to DNA breaks and facilitates the recruitment of DNA repair factors, such as BRCA1, by promoting PAR production [30, 31]. Given the essential role of PARP1 in DNA repair, we hypothesized that the mechanism by which FAO regulates DNA repair involves PARP1. To test this idea, we first examined whether FAO inhibition modulates PARP1 activity by measuring levels of PARylation under DNA-damaged conditions. We observed that the intensity of nuclear PAR was increased after IR exposure or ETS treatment (Fig. 3A and Supplementary Fig. S3A). Notably, the induction of PAR was substantially diminished in CPT1A knockdown cells (Fig. 3A and Supplementary Fig. S3A). When we blotted whole cell lysates with anti-PAR antibody, CPT1A knockdown decreased PAR production in response to IR exposure (Fig. 3B and Supplementary Fig. S3B). Near-identical results were observed in cells after ETS treatment (Supplementary Fig. S3C). We did not find a difference in PARP1 expression after CPT1A knockdown (Supplementary Fig. S3D). Next, the generality of PARP1 regulation by FAO was tested by using another DNA damaging agent, doxorubicin (DOX) (Supplementary Fig. S3E), indicating that the FAO-mediated PARP1 regulation upon DNA damage may be a general phenomenon.

PARP1 catalyzes PARylation not only on target proteins but also on itself [30]. To examine whether PARylation of PARP1 is affected by FAO, a FLAG-tagged PARP1 was expressed in cells (Supplementary Fig. S3F). We found that FAO inhibition by ETO treatment or CPT1A knockdown markedly suppressed the auto-PARylation of PARP1 upon DNA damage (Fig. 3C and Supplementary Fig. S3G). Moreover, in agreement with our model, octanoate treatment was able to rescue the reduced auto-PARylation of PARP1 or nuclear PAR intensity imposed by FAO inhibition (Fig. 3D, E), demonstrating that FAO plays a crucial role in promoting PARP1 activity after DNA damage.

Based on these results, we reasoned that FAO may contribute to DNA repair by regulating PARP1 activity. To examine this, we analyzed the γH2AX foci formation in cells in which PARP1 expression was reduced by using siRNA against PARP1 (Supplementary Fig. S3H). Similar to those observed after FAO inhibition, reduction of PARP1 significantly delayed the clearance of γH2AX foci and ETO treatment did not exhibit additional effects on DNA repair (Fig. 3F). Overall, these data demonstrate that FAO positively regulates PARP1 activity during the DNA repair process.

### FAO regulates DNA repair and PARP1 activity through acetyl-CoA

**Fig. 4:**
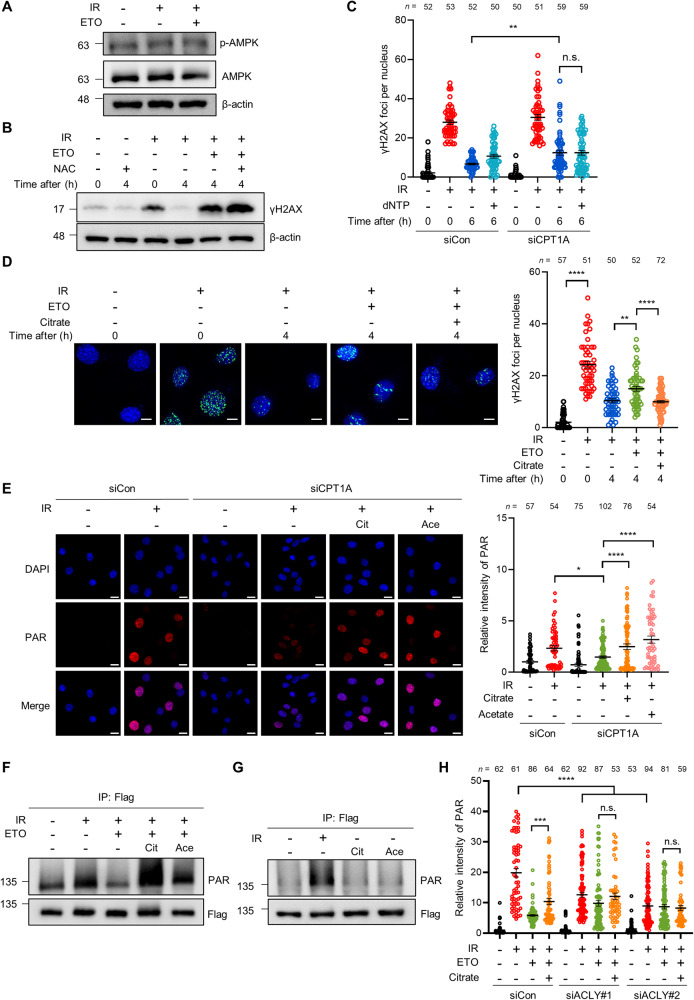
FAO adjusts PARP1 activity through acetyl-CoA upon DNA damage. **A** Immunoblot for p-AMPK and AMPK in the indicated cells. Immortalized MEFs were exposed to 3 Gy IR and then treated with or without ETO for 4 h. β-actin was used as loading control. **B** γH2AX protein levels in cells treated with the indicated drugs. Immortalized MEFs were exposed to 3 Gy IR and treated with or without ETO and/or 4 mM NAC for 4 h. **C** The number of γH2AX foci per nucleus of the indicated cells. Immortalized MEFs were transfected with siControl or with siRNAs against CPT1A. After transfection, cells were exposed to 3 Gy IR and treated with or without 100 μM dNTP for 6 h. Statistical analysis was performed using two-way ANOVA with Tukey’s multiple comparisons test. The number of cells analyzed per condition (n) is indicated. **D** Immunofluorescent staining was performed with a nuclear marker (DAPI) and anti-γH2AX antibody on cells. Immortalized MEFs were exposed to 3 Gy IR and then treated with or without 3 mM citrate for 4 h. Scale bar represents 6 μm. The number of γH2AX foci per nucleus as indicated (right). Statistical analysis was based on one-way ANOVA with Tukey’s multiple comparisons test. The number of cells analyzed is noted. **E** Representative images of immunofluorescence staining in control and CPT1A knockdown cells treated with the indicated drugs. Immunofluorescent staining was performed with nuclear marker (DAPI) and anti-PAR antibody on cells. Scale bar represents 20 μm. Relative fluorescence intensity of PAR as indicated (right). Statistical analysis was performed using one-way ANOVA with Tukey’s multiple comparisons test. The number of cells analyzed is indicated. **F, G** 293 T cells were transfected with pCMV-PARP1-3x Flag. After transfection, cells were treated with ETO, 3 mM citrate, and/or 50 mM acetate for 4 h as indicated and then irradiated with 10 Gy IR. Lysates were immunoprecipitated with Flag antibody and immunoblotted with anti-PAR antibody. **H** Relative fluorescence intensity of PAR in the indicated cells. Immortalized MEFs were transfected with siControl or two independent siRNAs against ACLY. After transfection, cells were treated with or without ETO and/or 3 mM citrate for 4 h and then irradiated with 3 Gy IR. Statistical analysis was performed using two-way ANOVA with Tukey’s multiple comparisons test. The number of cells analyzed per condition (n) is noted. In (C), (D), (E), and (H), the number of cells was pooled from three independent experiments. All error bars ± SEM. *p < 0.05, **p < 0.01 and ****p < 0.0001.

As our results showed that FAO potentiates DNA repair by promoting PARP1 activity, we next sought to examine the molecular mechanism. Given the importance of FAO in maintaining cellular energy homeostasis [11, 12], we first tested whether FAO inhibition impairs DNA repair by causing energy depletion. However, when we examined AMP-activated protein kinase (AMPK) activity by measuring its phosphorylation levels, we did not detect significant changes in FAO-inhibited cells (Fig. 4A). Consistent with these results, cellular ATP levels were not affected in ETO-treated cells after DNA damage (Supplementary Fig. S4A). FAO is also required for redox homeostasis by supporting the TCA cycle [32, 33]. Indeed, the TCA cycle intermediates, such as citrate and malate, are used to generate NADPH, providing the reducing force to sustain cellular reduced glutathione levels [32, 34]. Thus, we next investigated whether FAO affects DNA repair via reactive oxygen species (ROS). However, we found that FAO inhibited cells exhibited no significant changes in cellular ROS levels under our culture conditions (Supplementary Fig. S4B). This was further supported by the fact that antioxidant N-acetylcysteine (NAC) treatment did not rescue the delay of γH2AX reduction by FAO inhibition (Fig. 4B), indicating that ROS is not responsible for the FAO-mediated regulation of DNA repair. Recently, it was shown that fatty acids are essential for nucleotide synthesis in endothelial cells and thus CPT1A knockdown reduces the levels of pyrimidine and purine deoxynucleotides (dNTPs) [35]. Since failed dNTPs supplement could limit DNA repair, we investigated whether FAO supports DNA repair by promoting dNTPs synthesis. When the status of γH2AX foci was analyzed after DNA damage, supplementation of dNTPs did not rescue the impaired DNA repair by CPT1A knockdown (Fig. 4C). Thus, the impaired DNA repair by FAO suppression seemed not to be linked to energy homeostasis, ROS production and nucleotide synthesis.

FAO is a significant source of intracellular acetyl-CoA pool [11, 21]. Because FAO inhibition results in a reduction of cellular acetyl-CoA levels [36], we suspected that the mechanism by which FAO regulates DNA repair involves acetyl-CoA. To test this idea, we investigated whether acetyl-CoA replenishment facilitates the DNA repair process in FAO-inhibited cells. As citrate or acetate is catabolized into acetyl-CoA by ATP citrate lyase (ACLY) or acetyl-CoA synthetase 2 (ACSS2), respectively, we first treated cells grown under these conditions with the supplement of citrate. Importantly, citrate treatment appeared to restore the clearance of γH2AX foci in FAO-inhibited cells to a similar degree to that in control cells (Fig. 4D). The finding that FAO contributes to DNA repair by promoting PARP1 activity prompted us to examine whether acetyl-CoA could recover the decreased PARP1 activity by FAO inhibition. We found that citrate or acetate treatment was able to completely rescue PARP1 activity following CPT1A knockdown upon DNA damage (Fig. 4E). Moreover, the decreased auto-PARylation of PARP1 by FAO inhibition was restored in the presence of citrate or acetate (Fig. 4F), while both treatments without DNA damage had no effect on the PARylation of PARP1 (Fig. 4G). Additionally, we did not detect significant changes in cell cycle after citrate or acetate treatment (Supplementary Fig. S4C).

As FAO-derived citrate is exported to the cytoplasm and metabolized into acetyl-CoA by ACLY, we suspected that ACLY knockdown dampened the effects of citrate treatment. First, we found that knockdown of ACLY significantly inhibited levels of protein PARylation in a manner similar to FAO inhibition, and ETO treatment did not have addictive effects in ACLY knockdown cells (Fig. 4H and Supplementary Fig. S4D, E). Notably, whereas citrate treatment rescued the decreased nuclear PAR in control cells, it had no effect on PAR intensity in ACLY knockdown cells (Fig. 4H and Supplementary Fig. S4E). Collectively, these data support the idea that FAO regulates DNA repair and PARP1 activity via acetyl-CoA.

### FAO potentiates PARP1 activity by promoting its acetylation in response to DNA damage

**Fig. 5:**
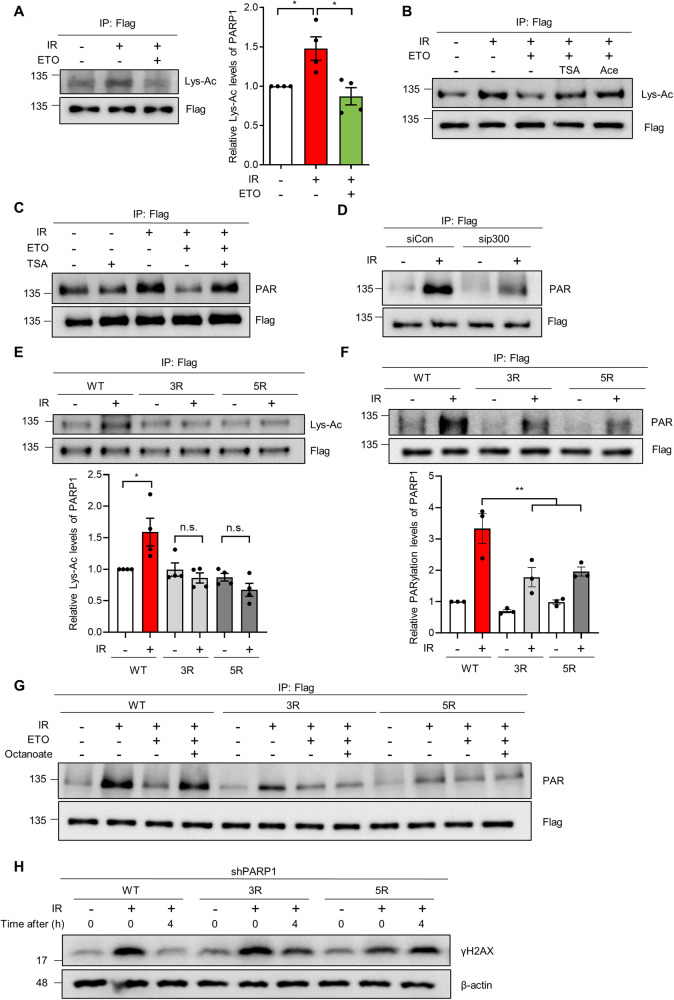
DNA damage-induced PARP1 acetylation affects its activity. **A** 293 T cells were transfected with pCMV-PARP1-3x Flag. Transfected cells were exposed to 10 Gy IR and treated with or without ETO for 4 h. Lysates were immunoprecipitated with Flag antibody and immunoblotted with anti-acetylated lysine (Lys-Ac) antibody. Relative PARP1 Lys-Ac levels as indicated (right). Statistical analysis was based on one-way ANOVA with Tukey’s multiple comparisons test. **B** 293 T cells were transfected with pCMV-PARP1-3x Flag. Transfected cells were treated with 0.3 μM TSA, 50 mM acetate, and/or ETO for 4 h before IR. Lysates were immunoprecipitated with Flag antibody and immunoblotted with anti-Lys-Ac antibody. **C** pCMV-PARP1-3x Flag transfected 293 T cells were treated with 0.3 μM TSA and/or ETO for 4 h and then irradiated 10 Gy IR. Lysates were immunoprecipitated with Flag antibody and immunoblotted with anti-PAR antibody. **D** Control or p300 knockdown 293 T cells, transfected with pCMV-PARP1-3x Flag, were exposed to 10 Gy IR. Lysates were immunoprecipitated with Flag antibody and immunoblotted with anti-PAR antibody. **E**, **F** 293 T cells were transfected with WT or two mutant forms of PARP1; 3 R (K498R/K521R/K524R) and 5 R (K498R/K505R/K508R/K521R/K524R). Transfected cells were exposed to 10 Gy IR. Lysates were immunoprecipitated with Flag antibody, followed by immunoblotting with anti-Lys-Ac and anti-PAR antibodies (upper panels). The immunoprecipitated Flag has been adjusted to be equal to make the levels of acetylated PARP1 (E) or PAR (F) comparable to those of immunoprecipitated Flag. Relative Lys-Ac and PARylation levels of PARP1 indicated (lower panels). Statistical analysis was performed using two-way ANOVA with Tukey’s multiple comparisons test. **G** 293 T cells were transfected with WT or two mutant forms of PARP1. Transfected cells were treated with or without ETO and/or 1 mM octanoate for 4 h and then treated with 10 μM PDD00017273 for 1 h before IR. Lysates were immunoblotted with anti-PAR antibody. **H** PARP1 knockdown 293 T cells were transfected with WT or two mutant forms of PARP1 and then irradiated with 10 Gy IR. Lysates were subjected to immunoblotting analysis with anti-γH2AX antibody. All error bars ± SEM. *p < 0.05 and **p < 0.01.

PARP1 can be acetylated by lysine acetyltransferase (KAT) p300 and CREB-binding protein (CBP), and acetylation of PARP1 enhances its PARylation activity [18, 37]. Given that fatty acids-derived acetyl-CoA is a major carbon source for acetylation of nuclear proteins [22] and that exogenous acetyl-CoA supplement rescues the reduction of PARP1 activity by FAO inhibition (Fig. 4), we reasoned that FAO may promote PARP1 activity by regulating its acetylation. First, to test whether FAO affects PARP1 acetylation upon DNA damage, we exposed cells, expressing a FLAG-tagged PARP1, to IR. PARP1 acetylation was determined after immunoprecipitation of PARP1 by immuno-blotting with anti-acetyl lysine antibody. We observed that IR exposure increased PARP1 acetylation levels, which was markedly inhibited by ETO treatment (Fig. 5A). The balance between KATs and lysine deacetylases (KDACs) is a major determinant of protein acetylation [38]. When cells were treated with pan KDAC inhibitors, such as trichostain A (TSA) or sodium butyrate (NaBu), both treatments were able to rescue the decreased PARP1 acetylation by FAO inhibition (Fig. 5B and Supplementary Fig. S5A). Consistent with our model, acetyl-CoA supplement by acetate or citrate treatment also rescued the decreased PARP1 acetylation in FAO inhibited cells, restoring its acetylation to control levels (Fig. 5B and Supplementary Fig. S5B).

We next investigated the significance of FAO-mediated regulation of PARP1 acetylation in its activity. When cells were treated with KDAC inhibitors, these treatments significantly restored the impaired auto-PARylation of PARP1 in FAO-inhibited cells (Fig. 5C and Supplementary Fig. S5C). Additionally, consistent with previous studies, when we inhibited p300 acetyltransferase activity by treating cells with C646, a p300 inhibitor, or by using siRNA against p300, the induction of PARylation of PARP1 was abrogated similar to the effect of FAO inhibition (Fig. 5D and Supplementary Fig. S5D, E), indicating that the regulation of PARP1 acetylation by FAO exerts an important role in PARP1 activity in response to DNA damage.

PARP1 was previously shown to be acetylated on five residues (K498, K505, K508, K521, and K524) by p300 [37]. Of note, mutations of three lysines (K498, K521, and K524) in PARP1 strongly suppressed auto-modification of PARP1 [39]. Thus, we sought to examine the contribution of these residues to the acetylation level and activity of PARP1 after DNA damage. As previously described [37], we mutated 3 (K498/K521/K524) or 5 (K498/K505/K508/K521/K524) lysine residues to arginine as a mimic of nonacetylated form of PARP1. When we assessed the acetylation level of these mutants, we observed that both 3R and 5R mutants were markedly less acetylated than wild-type PARP1 upon IR exposure (Fig. 5E). To determine the importance of acetylation of these residues for PARP1 activity, we examined PARylation levels of these mutants after IR exposure. Indeed, the auto-PARylation of PARP1 by DNA damage was significantly reduced in both mutants compared to wild-type (WT) PARP1 (Fig. 5F). Consistent with our previous results, octanoate treatment markedly rescued the reduced auto-PARylation of WT PARP1 after FAO inhibition, whereas it did not restore PARylation levels of PARP1 mutants (Fig. 5G). Lastly, in order to examine whether the acetylation of PARP1 is required for DNA repair, WT or PARP1 mutants were expressed in endogenous PARP1 knockdown cells (Supplementary Fig. S5F), and then assessed γH2AX expression upon DNA damage. We observed that reconstitution of cells with WT PARP1 can repair DNA damage after IR exposure, whereas the restoration of γH2AX expression was markedly delayed in cells reconstituted with acetylation mutants of PARP1 (Fig. 5H). Taken together, these data demonstrate that FAO promotes PARP1 acetylation after DNA damage, which accounts for a substantial contribution to PARP1 activity.

### FAO inhibition exacerbates DNA damage-induced genomic instability

**Fig. 6:**
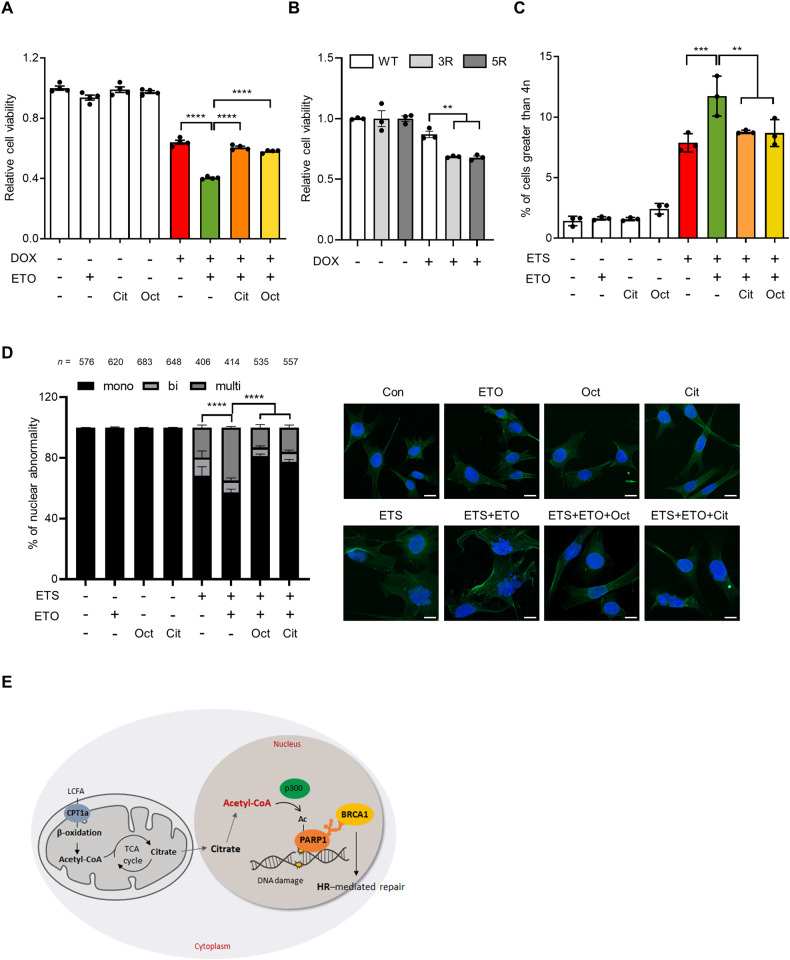
FAO is required to maintain genomic stability. **A** Cell viability of immortalized MEFs treated with the indicated drugs. Cells were treated with or without 0.05 μM DOX, 200 μM ETO, 0.5 mM citrate, and/or 50 μM octanoate for 48 h. Statistical analysis was based on one-way ANOVA with Tukey’s multiple comparisons test. **B** Cell viability of the 293 T cells, reconstituted with WT or two mutant forms of PARP1, treated with DOX. Statistical analysis was performed using two-way ANOVA with Tukey’s multiple comparisons test. **C** Immortalized MEFs were treated with or without 10 μM ETS, 200 μM ETO, 0.1 mM citrate, and/or 50 μM octanoate for 15 h. The following day, growth media was replaced with fresh media. Two days later, cells were stained with propidium iodide and analyzed by flow cytometry. Statistical analysis was performed using one-way ANOVA with Tukey’s multiple comparisons test. **D** Percentages of multinucleated, binucleated, and mononucleated cells in the indicated cells. Immortalized MEFs were treated with ETS, ETO, citrate and/or octanoate as indicated. The number of cells pooled from three independent experiments is indicated. Statistical analysis was performed using two-way ANOVA with Tukey’s multiple comparisons test. The indicated p-values represent comparison within multinuclei. Representative images of cells staining with DAPI and anti-phalloidin antibody as indicated (right). Scale bar represents 20 μm. **E** A proposed model for role of FAO in DNA repair. All error bars ± SEM. **p < 0.01, ***p < 0.001 and ****p < 0.0001.

Defects in DNA repair may lead to accumulation of DNA damage and genome instability [6]. Our results demonstrated that FAO contributes to HR repair by promoting PARP1 activity. To assess the functional relevance of the FAO-mediated regulation of DNA repair, we first examined the ability of FAO to determine the cellular sensitivity to DNA damage. Given the importance of FAO in facilitating DNA repair, we speculated that FAO inhibition may sensitize cells to DNA damage. To test this idea, we treated cells with DOX, a topoisomerase 2 inhibitor that causes DSBs and has been shown to induce cell death across multiple cell lines [40]. Importantly, we observed that ETO treatment potently synergized with DOX to reduce cell viability (Fig. 6A), indicating that cells are more sensitive to DNA damage when FAO is impaired. Notably, acetyl-CoA supplement by citrate or octanoate treatment blunted this synergistic effect (Fig. 6A). Similar to DOX treatment, both ETO treatment or CPT1A knockdown significantly decreased cell viability following exposure to IR, which was rescued by the supplementation of acetyl-CoA (Supplementary Fig. S6A, B). Moreover, we found that cells reconstituted with PARP1 acetylation mutants (Supplementary Fig. S5F) were more sensitive to DNA damage in comparison with cells reconstituted with WT PARP1 (Fig. 6B). These data provide further support for the important role of FAO in PARP1 activation and DNA repair upon DNA damage.

We next assessed whether FAO inhibition affects genome integrity after genotoxic stress. An impaired HR repair during G2/M phase can lead to polyploidy. Indeed, more polyploidy cell populations were observed in ETO-treated cells compared to untreated cells after DNA damage, which was rescued by citrate or octanoate treatment (Fig. 6C). We next examined the chromosome abnormalities after DNA damage by staining nucleus and F-actin, respectively, with DAPI and fluorescent phalloidin. The number of binucleated or multinucleated cells was elevated in FAO-inhibited cells after ETS treatment (Fig. 6D). We also obtained comparable results in cells after IR exposure (Supplementary Fig. 6C, D). In line with our hypothesis, acetyl-CoA supplement reversed this phenotype (Fig. 6D). Collectively, these results provide evidence that FAO is required for the proper DDR, particularly in DNA repair and that loss of this critical metabolic pathway leads to genomic instability after DNA damage.

## Discussion

In this study, we define an important role of FAO in cellular response to DNA damage by promoting DNA repair. Our study shows that FAO inhibition abrogates DNA repair, particularly HR repair. Mechanistically, FAO participates in DNA repair by potentiating PARP1 activity. We discover that FAO facilitates DNA damage-induced PARP1 acetylation via acetyl-CoA, which contributes to the PARylation activity of PARP1. As a consequence, FAO suppression aggravates genomic instability in response to genotoxic stress (Fig. 6E).

Recent studies have shed light on potential implication of FAO in many cellular processes by modulating protein acetylation. FAO-derived acetyl-CoAs not only serve to replenish mitochondrial acetyl-CoA pool, but also export to the cytosol as a form of citrate. Citrate, transported by citrate carrier, is catabolized to acetyl-CoA by ACLY and then fulfills cytosolic and nuclear acetyl-CoA pool. Several groups have provided evidence that FAO-derived acetyl-CoA is essential for acetylation of mitochondrial and cytosolic proteins [23, 36]. Moreover, it was shown that FAO can occupy most of the total acetyl-CoA content for nuclear histone acetylation, even in the presence of glucose [22]. Interestingly, our findings are in line with a recent work reporting that facilitating acetyl-CoA production from citrate by nuclear ACLY is required for BRCA1 recruitment and HR repair [41]. In this study, we propose that FAO is required for HR repair by modulating PARP1 acetylation. We demonstrate that DNA damage promotes PARP1 activity by increasing its acetylation, which is impaired by FAO inhibition. We also show that exogenous citrate or acetate can restore PARP1 activity in FAO inhibited cells. Thus, our study and others suggest that FAO could have important roles in various cellular signaling pathways by modulating protein acetylation.

Although our data highlights the crucial role of PARP1 acetylation at K498, K505, K508, K521, and K524 lysine residues in regulating PARP1 activity and DSB DNA repair, it does not preclude the involvement of acetylation of other lysine residues of PARP1. Indeed, over 20 acetylation sites have identified on PARP1 [17]. Moreover, it was previously shown that microrchidia family CW-type zinc finger 2 (MORC2), a chromatin remodeling enzyme, induces acetylation of PARP1 at lysine 949 by acetyltransferase NAT10 upon DNA damage, which inhibits ubiquitin-dependent PARP1 degradation and contributes to recruitment of repair proteins to damaged DNA [18]. On the other hand, PARP1 is implicated in various repair pathways, such as single-strand break DNA repair, and also has diverse functions in the cell [30, 42]. Thus, it will be important for future work to examine how these lysine residues of PARP1 are precisely modified to regulate genome stability and to contribute to other roles of PARP1.

Our current study reveals the profound impact of FAO on DNA repair. However, as the DDR is a highly orchestrated and intertwined signaling response pathway [1, 5], it is possible that FAO may be important for other components of the DDR, such as cell cycle arrest and cell death. Indeed, it has been reported that FAO affects cellular sensitivity to genotoxic stress [15]. We also observed that FAO inhibition sensitizes cells to DNA damage (Fig. 6A). In addition, FAO inhibition appears to arrest cells at the G2/M phase after DNA damage (Fig. 2D). Thus, to what extent the essentiality of FAO in DNA repair can be extrapolated to other branches of the DDR needs to be evaluated, but the fact that FAO appears to function as a significant metabolic regulator in cellular response to DNA damage suggests that it will be important for future studies to examine how FAO coordinately regulates cell survival and genomic fidelity after genotoxic stress.

Alteration in lipid metabolism is one of the key metabolic phenotypes of senescence or aging [43, 44]. It is important to notice that aberrant fatty acid utilization is intimately associated with age-related disease such as non-alcoholic fatty liver disease, chronic kidney disease and sarcopenia [45, 46]. However, it remains largely unknown whether defective fatty acid metabolism is causative of aging. Our data demonstrates that the perturbation of FAO leads to delayed DNA repair by suppressing PARP1 activity, resulting in DNA damage-induced genomic instability. Given the in vivo accelerated aging phenotypes of patients with defects in DNA repair [7], in addition to the essential role of PARP1 in the DDR and cellular senescence [30, 47], we propose that dysregulation of FAO might contribute to senescence or aging processes, in part by limiting cellular DNA repair capacity. On the other hand, several lines of evidence have shown that obesity drives accumulation of senescent cells in adipose tissue as well as other organs and obese patients have a higher risk of developing various age-related diseases [47, 48]. As abnormal fat accumulation in obese or diabetic mice often accompanies with impaired fatty acid utilization [49], it is plausible that dysregulation of mitochondrial FAO could be involved in obesity-related cellular senescence and diseases.

Communication between the mitochondria and nucleus is essential for maintaining proper cellular function. Key players involving in nuclear-mitochondria signaling, including PARP1, SIRT1, PGC-1α, and AMPK, have been shown to play critical roles in regulating mitochondrial functions [50]. For example, PARP1 activation upon DNA damage leads to NAD^+^ depletion, which subsequently inhibits SIRT1 activity [51]. The loss of SIRT1 function results in mitochondrial dysfunctions, such as increased ROS production. In this study, we provide compelling evidence supporting the role of mitochondrial FAO in promoting DNA repair and maintaining genomic stability within the nucleus. We demonstrate that FAO plays a crucial role by supplying acetyl-CoA, which is necessary to promoting PARP1 activity. Collectively, our findings, along with previous studies, underscore the importance of nuclear-mitochondrial communications in preserving cellular homeostasis. Elucidating the mechanisms that orchestrate this crosstalk might be crucial for understanding genome instability, mitochondrial dysfunction and their associations with tumorigenesis and aging.

In sum, our studies illustrate that FAO is an essential component in the DDR, in part by regulating DNA repair. These findings suggest that the regulation of cell metabolic response to DNA damage could provide an important area for understanding of cellular stress response and aging.

## Materials and methods

### Cell culture

Mouse Embryonic Fibroblast (MEFs), Human embryonic kidney 293 (HEK293), HEK293T, and HeLa cells were cultured in Dulbecco’s modified Eagle’s medium (Welgene, Gyeongsan, Republic of Korea, LM001-07) supplemented with 10% fetal bovine serum (Gibco, Grans Island, NY, USA, 1600-044) and penicillin streptomycin (Biowest, Nuaille, France, L0022-100).

### Expression Vectors

pCBASceI plasmid (26477), DSB reporter system plasmid (98895), mCherry HR donor plasmid (98896), and pCMV PARP1-3x-Flag (111575) were purchased from addgene. PARP1 mutants were generated using mutagenesis kit (Agilent, Santa Clara, CA, USA, 200523) according to the manufacturer’s instructions. Primer sequences for mutagenesis are listed in Supplementary Table 1.

### siRNA and shRNA

10 or 20 nM siRNAs were transfected in cells using lipofectamine RNAiMAX (Invitrogen, Carlsbad, CA, USA) according to the manufacturer’s protocols. siRNA sequences are listed in Supplementary Table 2. shPARP1 (SHCLNGTRCN0000007928) was purchased from Sigma-Aldrich.

### Real-time PCR

Total RNA preparation, reverse transcription, and real time PCR were performed as previously reported [52]. Primer sequences were: TTGATCAAGAAGTGCCGGACGAGT and GTCCATCATGGCCAGCACAAAGTT for mouse *Cpt1a*; AGGAAGTGCCACCTCCAACAGT and CGCTCATCACAGATGCTGGTCA for mouse *Acly*; TCAATTTTAAGACCTCCCTGTGG and TGAATTCATACCAGAGCCACC for human *PARP1* (3’UTR); AGAGAAAAGGCGATGAGGTG and TTAGCTCGTCCTTGATGTTCC for human *PARP1* (CDS); AGCCATGTACGTAGCCATCC and CTCTCAGCTGTGGTGGTGAA for mouse *β-actin*.

### ATP measure

Cells were plated into 96-well plates at 1000 cells per well in 100 μl media. The following day, cells were exposed to 3 Gy IR and then treated with or without etomoxir for 4 h. ATP levels were detected using Cell Titer GLO (Promega, Fitchburg, WI, USA, G7571) according to the manufacturer’s instructions.

### DNA repair reporter

For DNA repair reporter analysis, 293 cells were infected with DNA repair reporter and selected by G418 (600 μg/ml). Infected 293 cells were transfected with 1 μg pDonor mCherry HR plasmid and 2.5 μg pCBASceI plasmid using lipofectamine 3000. Forty-eight hours after transfection, cells were trypsinized, resuspended in PBS, and analyzed by flow cytometry (BD Bioscience, San Jose, CA, USA, FACS Aria Fusion). Acquired data were analyzed using FlowJo software.

### Western blot

Cells were lysed in RIPA lysis buffer (ATTO, Tokyo, Japan, WSE-7420 EzRIPA lysis Kit or Cell Signaling Technology, Danvers, MA, USA, 9806s) supplemented with protease inhibitor and phosphatase inhibitor cocktail (ATTO, WSE-7420 EzRIPA lysis Kit). Cell lysates were separated by sodium dodecyl sulfate-polyacrylamide gel electrophoresis and transferred to nitrocellulose membrane (GE Healthcare, Buckinghamshire, UK, 10600001). Membranes were blocked with 5% nonfat milk and incubated with primary antibodies overnight at 4 °C. The next day, blots were incubated with secondary antibodies for 1 h at room temperature (RT) and detected using LAS4000. Uncropped immunoblots are included in Supplementary information.

### Immunoprecipitation

Cells were lysed in RIPA buffer (50 mM Tris-HCl [pH 7.4], 150 mM NaCl, 1% NP-40, 0.25% deoxy sodium chlorate, 1 mM EDTA) and sonicated three times for 5 sec each. Lysates were immunoprecipitated with Flag M2 Magnetic beads (Sigma-Aldrich, St. Louis, MO, USA, M8823) overnight at 4 °C on a rotating platform. After washing 3 times with TBS buffer (50 mM Tris HCl [pH 7.4], 150 mM NaCl), the bound proteins were eluted by Flag peptide (Sigma-Aldrich, F3290) and heated to 95 °C for 10 min in sodium dodecyl sulfate (SDS) loading buffer. Immunoblotting analysis was carried out with Flag antibody (Sigma-Aldrich, F1804) and anti-PAR-monoclonal antibody (Trevigen, Gaithersburg, MD, USA, #4335-MC-100).

To detect PARP1 acetylation, we performed a denaturation immunoprecipitation [53]. 293 T cells were transfected with pCMV-PARP1-3x Flag for 48 h. Cells were lysed with 100 μl of denaturing buffer (1% SDS, 5 mM EDTA, 10 mM β-mercaptoethanol) and boiled for 10 min. The lysates were mixed with 900 μl of denaturing RIPA buffer (1% NP40, 150 mM NaCl, 50 mM Tris-HCl [pH 8.0], 0.5% sodium deoxycholate, 0.1% SDS) supplemented with protease inhibitors. The lysates were collected for pre-clearing with 50% Sepharose G beads (Cytiva, Tokyo, Japan, 17061801) for 1 h at 4 °C. After centrifugation, the supernatants were incubated with Flag antibody containing sepharose G beads overnight at 4 °C. After washing 3 times with denaturing RIPA buffer, the bound proteins were eluted by Flag peptide and boiled for 10 min in SDS loading buffer. The supernatants were used for western blot. Immunoblotting analysis was performed with Flag antibody and anti-acetylated lysine antibody (Cell Signaling Technology, 9441 s).

### Detection of PARylated proteins

PARylated proteins were detected as described previously with slight modification [54]. Cells were treated with 10 μM PDD00017273, PARG inhibitor, for 1 h before irradiation to sustain PAR levels. Also, PDD00017273 was added to the lysis buffer for all PARylation-related experiments. Cells were lysed in PAR buffer (50 mM Tris-HCl [pH 7.5], 400 mM NaCl, 1 mM EDTA, 1% NP-40, 0.1% sodium deoxycholate) and sonicated three times for 5 sec each. Lysates were used for immunoblotting.

### Immunoflourescence

Cells were cultured on coverslips in 6 cm dishes. Cells were fixed in 4% paraformaldehyde for 5 min at RT. After PBS washing, cells were permeabilized for 20 min on PBS containing 0.5% Triton X-100. The permeabilized cells were blocked in 10% normal goat serum (NGS) for 1 h. Then the cells were incubated with the indicated antibody in 0.1% PBST with 10% NGS overnight at 4 °C. After washing with 0.1% PBST, cells stained with Alexa 594, FITC, or Alexa 488 antibody for 1 h at RT. Finally, cells were washed and mounted with Vectashield mounting medium with DAPI (Vector Laboratory, Burlingame, CA, USA, H-1200). Fluorescence images were captured using confocal microscope (Carl Zeiss Oberkochen, Germany, LSM800) or DeltaVision Cell imaging system (PersonalDV, Applied Precision/GE Healthcare, USA).

### Comet assay

Cells were suspended in PBS and combined with molten low melting agarose (Trevigen, 4250-050-02) at a ratio of 1:10. The cell-agarose mixture was immediately spread onto a microscope slide. Each slide was placed flat for 4 h at 4 °C in the dark and then immersed in lysis solution for 30 min. The samples were electrophoresed at 25 V for 30 min and stained with Vectashield mounting medium with DAPI according to the manufacturer’s instructions. Slides were captured by fluorescence microscopy and analyzed using Comet Score V 2.0 software (TriTek Corp, Sumerduck, VA, USA).

### Cell viability assay

Cells were plated into 96-well plates at 3,000 or 5,000 cells per well in 100 μl of media. The following day, cells were treated with or without 0.05 μM DOX, 200 μM ETO, 0.5 mM citrate and 50 μM octanoate or exposed to 10 Gy IR in the presence or absence of ETO, citrate and octanoate. After 48 h treatment, cell viability was analyzed using Cell Titer GLO (Promega, Fitchburg, WI, USA, G7571) or Cell Counting Kit-8 ((Dojindo Molec Tech, SKU, CD04) per the manufacturer’s instruction. The absorbance was measured at 450 nm using a microplate reader.

### Antibody and reagents

Antibody: β-actin (Genetex, Irvine, CA, USA, GTX109639), CPT1A (Abcam, Cambridge, UK, ab128568), PARP1 (Abcam, ab32138), PAR (Trevigen, 4335-MC-100, 4336-BPC-100), rH2AX (Merck Millipore, Bedford, MA, USA, 05-636 and Cell Signaling Technology, 2577 s), Flag M2 (Sigma-Aldrich, F1804), Lys-Ac (Cell Signaling Technology, 9441), 53BP1 (Cell Signaling Technology, 4937 s), BRCA1 (Santa Cruz Biotechnology, California, USA, sc-6954), AMPK (Cell Signaling Technology, 5831 s), p-AMPK (Cell Signaling Technology, 2535 s), Alexa 594 (Invitrogen, A11012), FITC (Santa Cruz Biotechnology, sc-516140), Alexa 488 (Invitrogen, A11029), Alexa Fluor™ 488 Phalloidin (Thermo Fisher Scientific, A12379), Mouse IgG-HRP (Genetex, GTX213111-01), and Rabbit IgG-HRP (Genetex, GTX213110-01). Trichostatin A (T8552), Sodium Butyrate (B5887), Etomoxir (E1905), Etoposide (E1383), Doxorubicin (D1515), C646 (SML0002), Octanoate (C5038), Sodium Acetate (S5636), and Sodium Citrate (PHR1416) were purchased from Sigma-Aldrich. PDD00017273 (Selleckchem Chemicals, Houston, Texas, USA, S8862) and Nocodazole (S2775) were purchased from Selleckchem.

### Analysis of nuclear abnormality

Cells were treated with or without 10 μM ETS, 200 μM ETO, 0.5 mM citrate, and/or 50 μM octanoate for overnight or exposed to 3 or 5 Gy IR in the presence of ETO for 48 h. Cells were fixed in 4% paraformaldehyde for 5 min, permeabilized for 20 min in 0.5% PBST, and blocked in 10% normal goat serum (NGS) for 1 h. After phalloidin (Thermo Fisher Scientific, A12379) staining, cells were mounted with Vectashield mounting medium with DAPI. Phalloidin and DAPI visualize F-actin filaments and the nucleus, respectively. Based on the DAPI-stained nuclei, the presence of micronuclei or extensive nuclear blebbing was defined as nuclear abnormalities, and the cells were classified into mono-, bi-, and multi-nucleus according to the number of nuclei.

### Statics and analysis

All experiments were performed independently at least three times with similar results. Sample numbers and sizes are indicated by dot, and the n number is noted on each figure. All statistical analyses and P values are described in the figure legends. Unpaired two-tailed Student’s t-test was used to compare the two groups. One-way ANOVA with Tukey’s multiple comparisons test was performed to compare more than two groups. Two-way ANOVA was used to compare two independent variables, followed by Tukey’s multiple comparisons test. All immunofluorescence images and immunoblots are representative of at least three independent experiments with comparable results obtained.

## Supplementary information

Supplementary Table 1

Supplementary Table 2

Reproducibility checklist

full and uncropped western blots

Supplementary Figures

## Data Availability

The data generated and analyzed in the current study are available within the manuscript. Additional data are available from the corresponding author upon reasonable request.
