# Supplementary material for: Fatty acid oxidation facilitates DNA double-strand break repair by promoting PARP1 acetylation
Source: Cell Death Dis. 2023 Jul 15;14(7):435. doi: 10.1038/s41419-023-05968-w (PMC10349888; doi:10.1038/s41419-023-05968-w)
Supplement: Supplementary file 1 — Supplementary Table 1 [file 41419_2023_5968_MOESM1_ESM.docx]

**Table S1. Primers for cloning**

| \| Plasmids \| Primers \| Sequence (5' to 3') \| \| --- \| --- \| --- \| \| pCMV-PARP1-3x Flag K498R \| Forward \| GTTGTGGCCCCAAGAGGGAGGTCAGGGGCTGCGCTCTC \| \| Reverse \| GAGAGCGCAGCCCCTGACCTCCCTCTTGGGGCCACAAC \| \| pCMV-PARP1-3x Flag K505R \| Forward \| CAGGGGCTGCGCTCTCCAGAAAAAGCAAGGGCCAGGTC \| \| Reverse \| GACCTGGCCCTTGCTTTTTCTGGAGAGCGCAGCCCCTG \| \| pCMV-PARP1-3x Flag K508R \| Forward \| CTCTCCAAAAAAAGCAGGGGCCAGGTCAAGGAG \| \| Reverse \| CTCCTTGACCTGGCCCCTGCTTTTTTTGGAGAG \| \| pCMV-PARP1-3x Flag K521R \| Forward \| GTATCAACAAATCTGAAAGGAGAATGAAATTAACTC \| \| Reverse \| GAGTTAATTTCATTCTCCTTTCAGATTTGTTGATAC \| \| pCMV-PARP1-3x Flag K524R \| Forward \| CAAATCTGAAAAGAGAATGCGATTAACTCTTAAAGGAGGAG \| \| Reverse \| CTCCTCCTTTAAGAGTTAATCGCATTCTCTTTTCAGATTTG \| \| pCMV-PARP1-3x Flag K521R/524R \| Forward \| GTATCAACAAATCTGAAAGGAGAATGCGATTAACTC \| \| Reverse \| GAGTTAATCGCATTCTCCTTTCAGATTTGTTGATAC \| \| pCMV-PARP1-3x Flag K505R/K508R \| Forward \| CTCTCCAGAAAAAGCAGGGGCCAGGTCAAGGAG \| \| Reverse \| CTCCTTGACCTGGCCCCTGCTTTTTCTGGAGAG \| |  |  |
| --- | --- | --- | --- | --- | --- | --- | --- | --- | --- | --- | --- | --- | --- | --- | --- | --- | --- | --- | --- | --- | --- | --- | --- | --- | --- | --- | --- | --- | --- | --- | --- | --- | --- | --- | --- | --- | --- | --- | --- | --- |
|  |  |  |
|  |  |  |
|  |  |  |
|  |  |  |
|  |  |  |
|  |  |  |
|  |  |  |
|  |  |  |
|  |  |  |
|  |  |  |
|  |  |  |
|  |  |  |
|  |  |  |
|  |  |  |
|  |  |  |
|  |  |  |
|  |  |  |
|  |  |  |
