# Supplementary material for: Fatty acid oxidation facilitates DNA double-strand break repair by promoting PARP1 acetylation
Source: Cell Death Dis. 2023 Jul 15;14(7):435. doi: 10.1038/s41419-023-05968-w (PMC10349888; doi:10.1038/s41419-023-05968-w)
Supplement: Supplementary file 2 — Supplementary Table 2 [file 41419_2023_5968_MOESM2_ESM.docx]

**Table S2. List of siRNAs used in this study**
