# Supplementary material for: Fatty acid oxidation facilitates DNA double-strand break repair by promoting PARP1 acetylation
Source: Cell Death Dis. 2023 Jul 15;14(7):435. doi: 10.1038/s41419-023-05968-w (PMC10349888; doi:10.1038/s41419-023-05968-w)

Figure 1 A

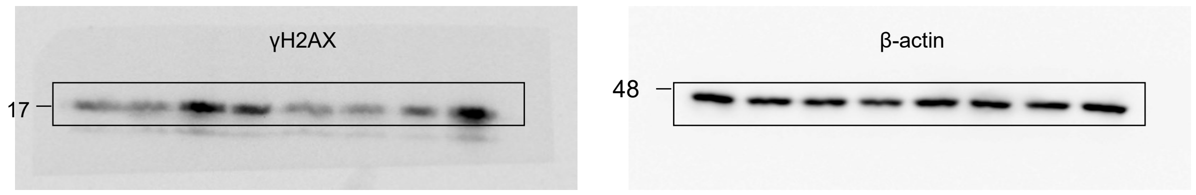

Figure 1 F

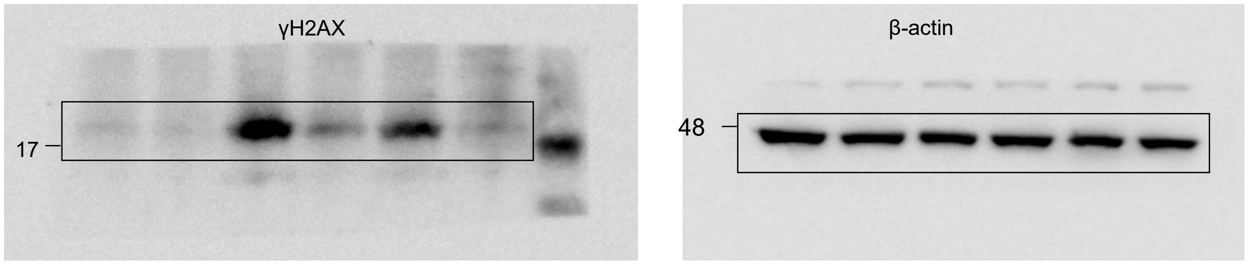

Figure 2 E

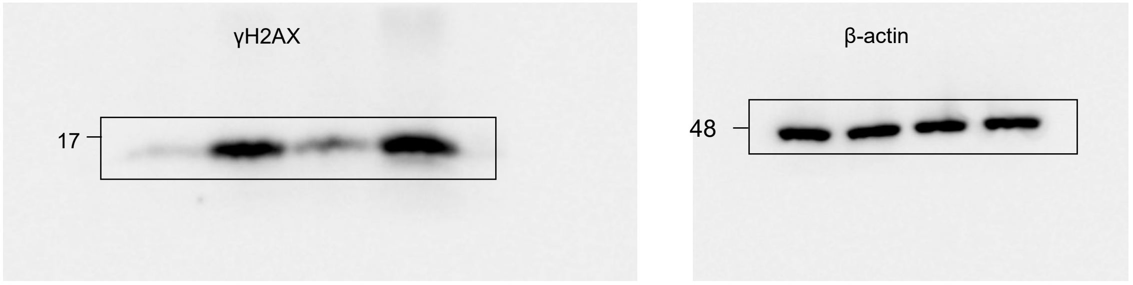

Figure 3 B

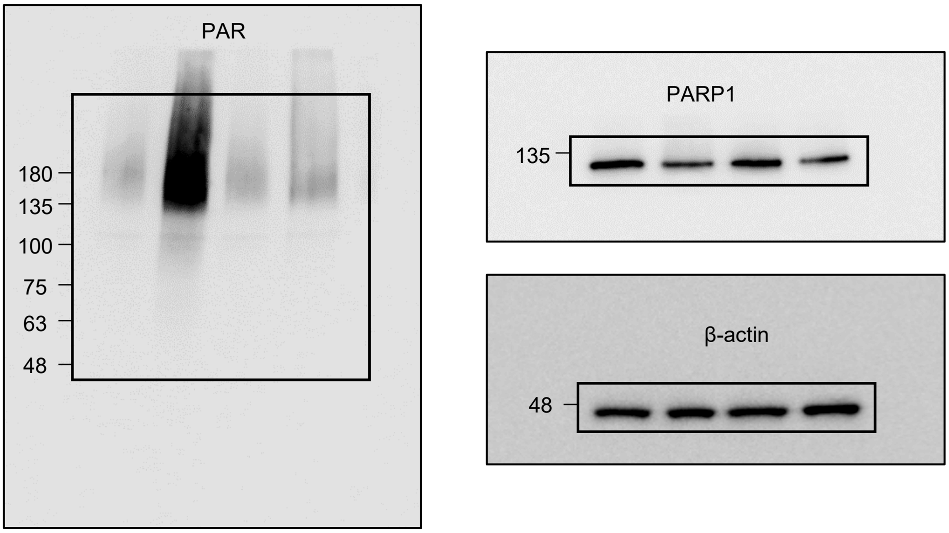

Figure 3 C

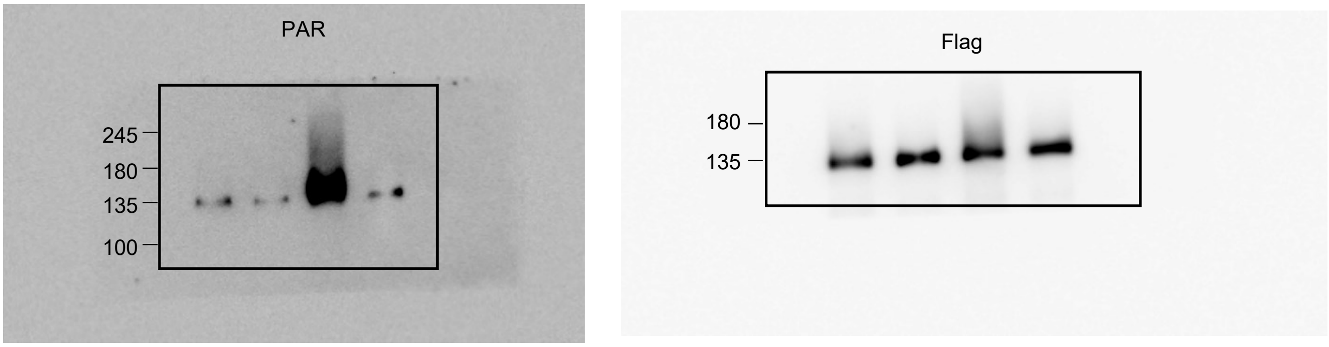

Figure 3 D

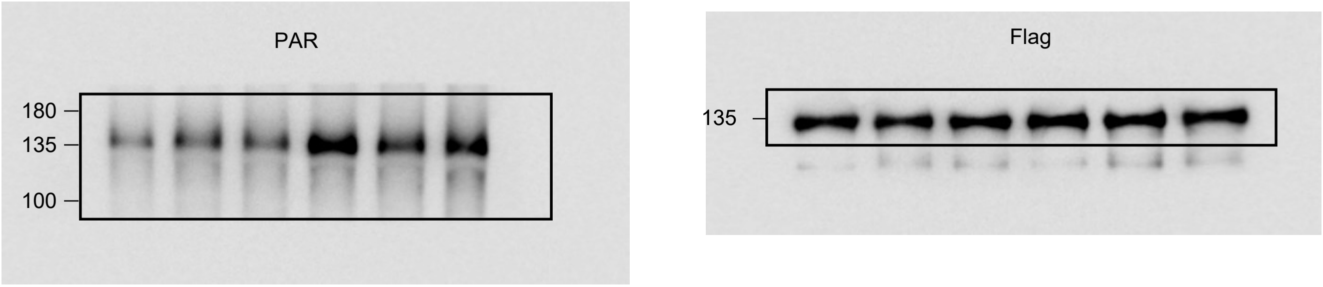

Figure 4 A

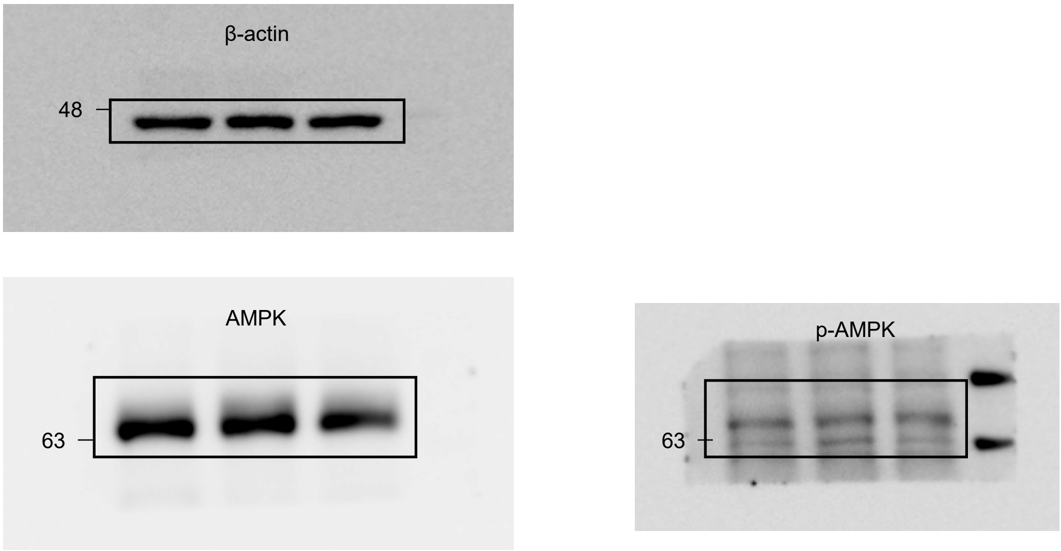

Figure 4 B

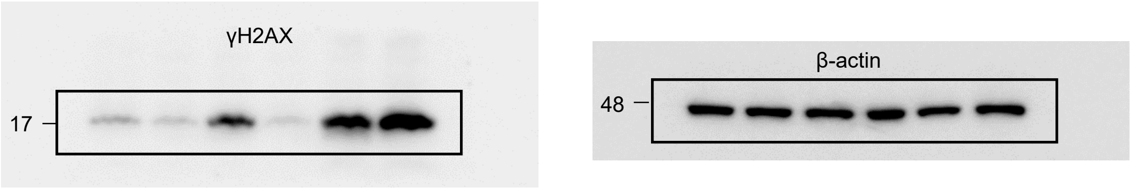

Figure 4 F

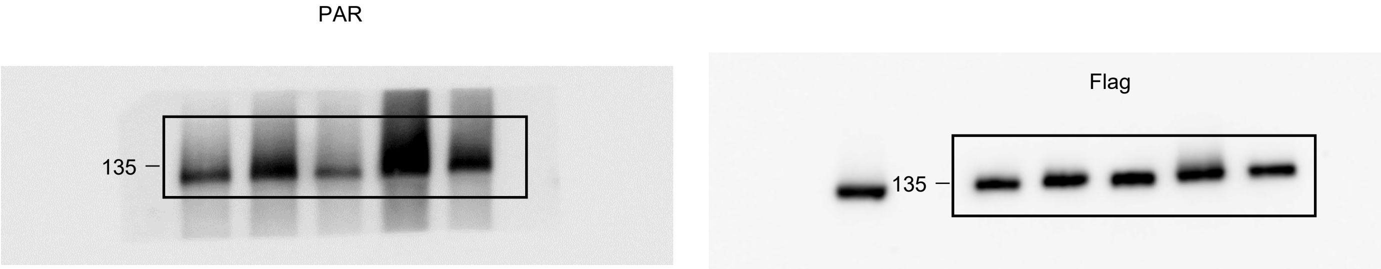

Figure 4 G

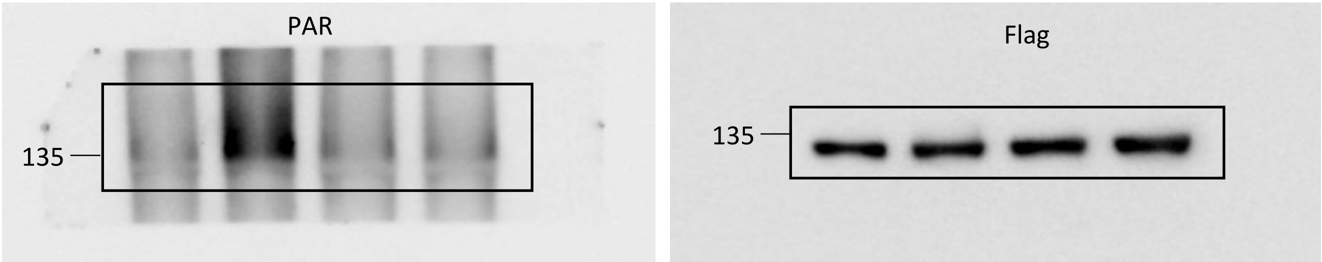

Figure 5 A

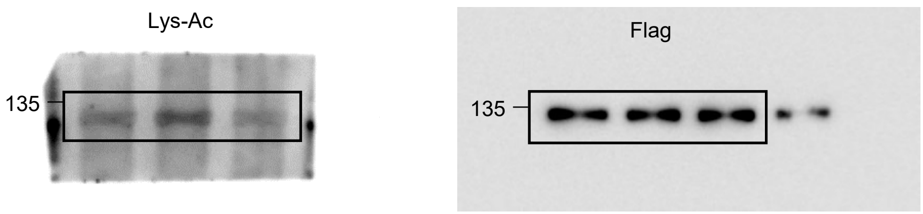

Figure 5 B

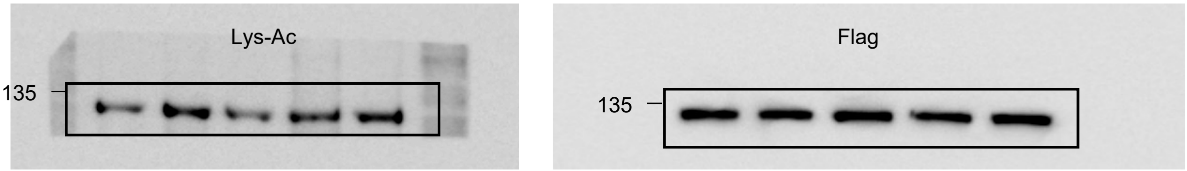

Figure 5 C

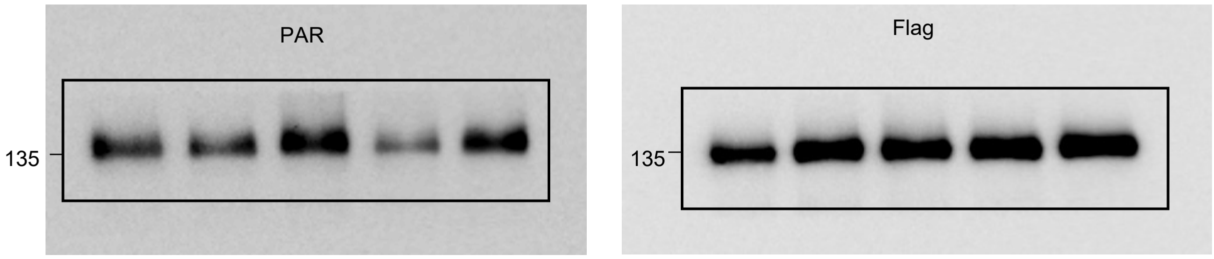

Figure 5 D

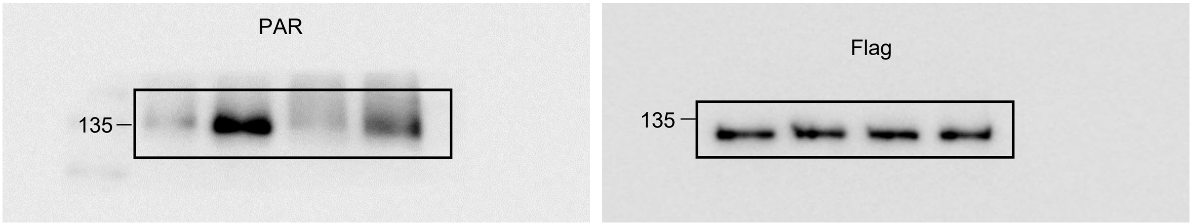

Figure 5 E

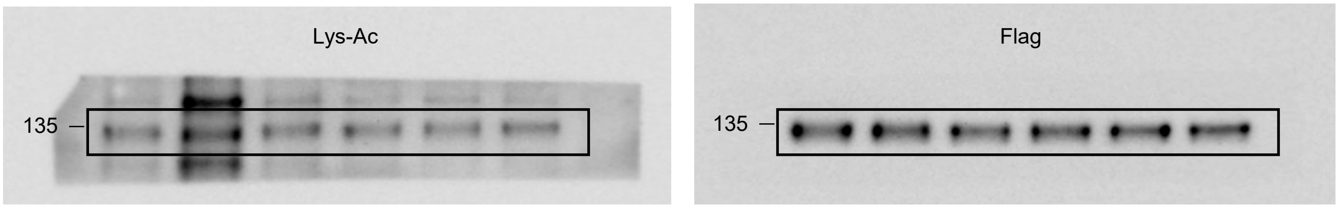

Figure 5 F

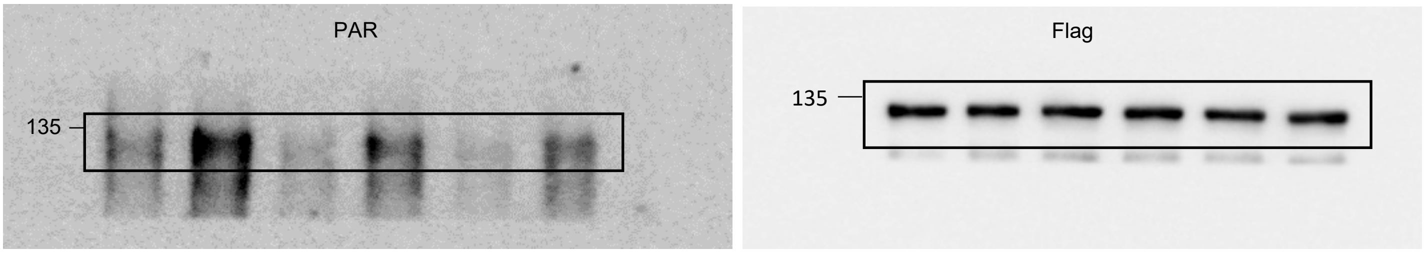

Figure 5 G

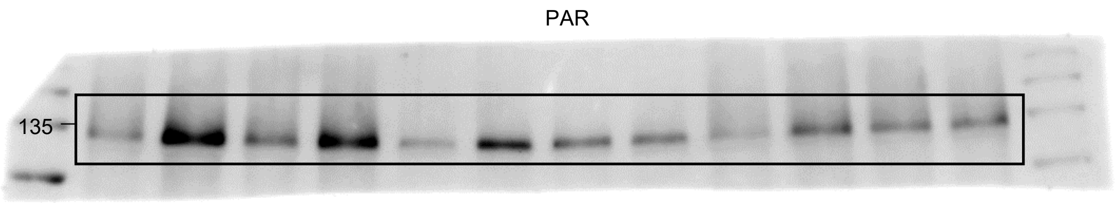

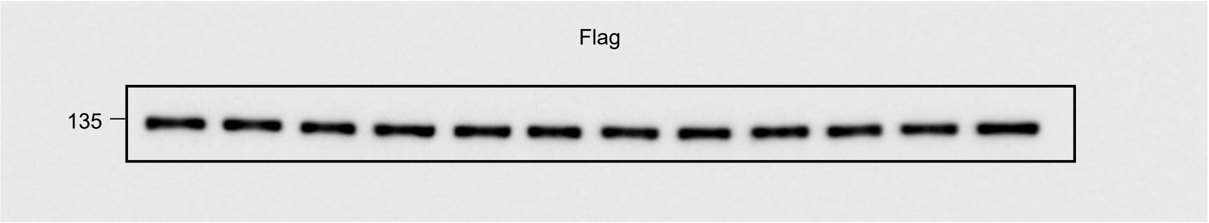

Figure 5 H

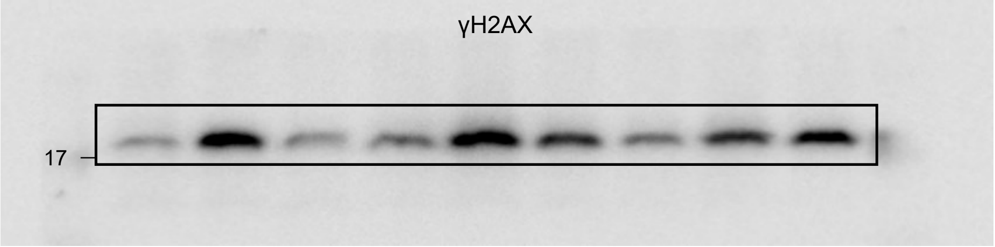

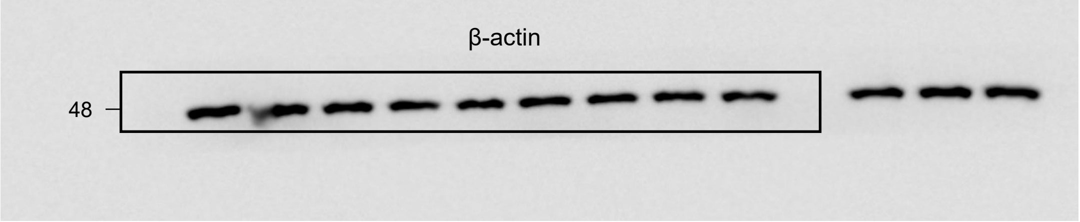

Supplemantary Figure 1 C

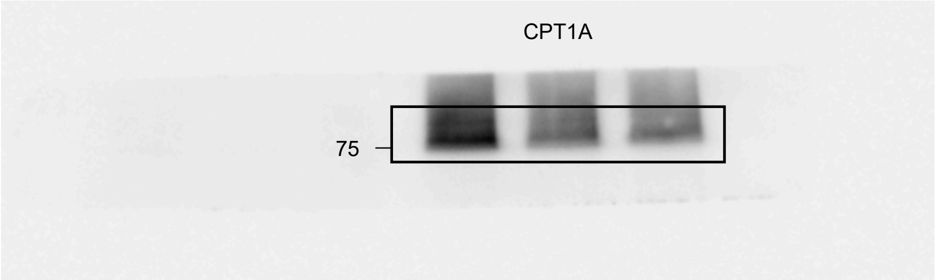

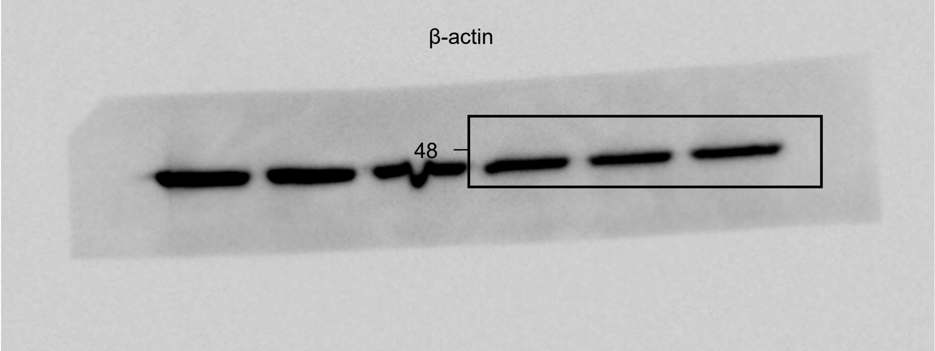

Supplemantary Figure 1 D

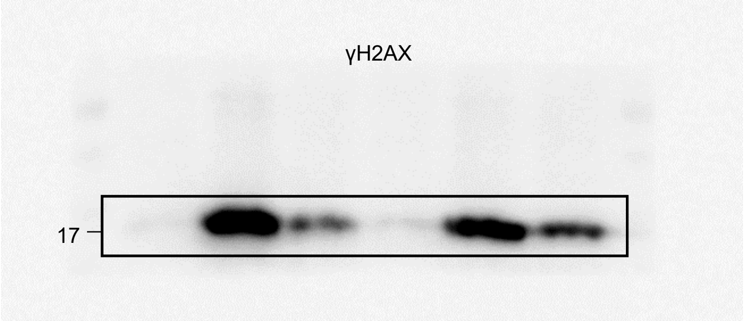

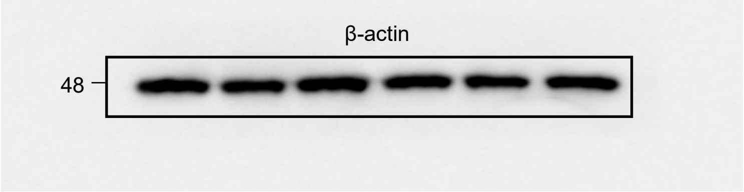

Supplemantary Figure 1 E

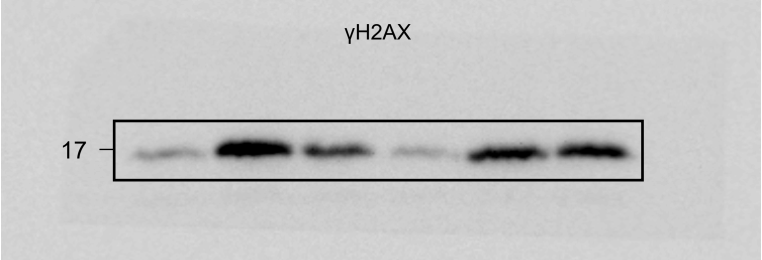

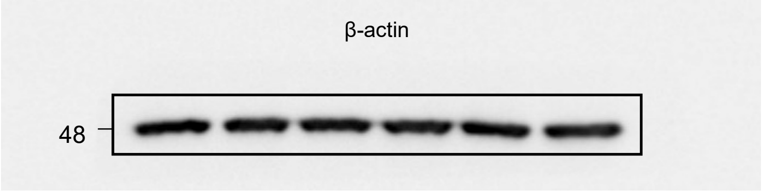

Supplemantary Figure 3 C

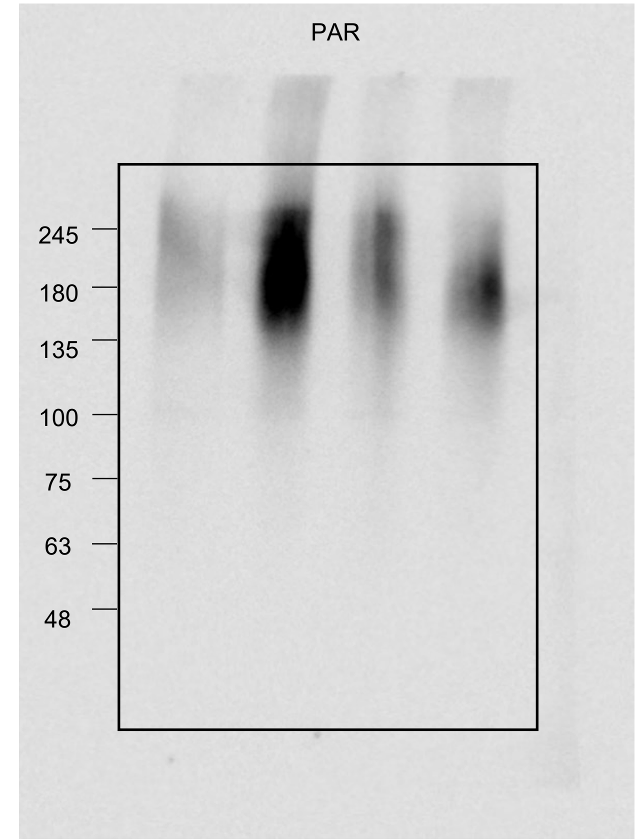

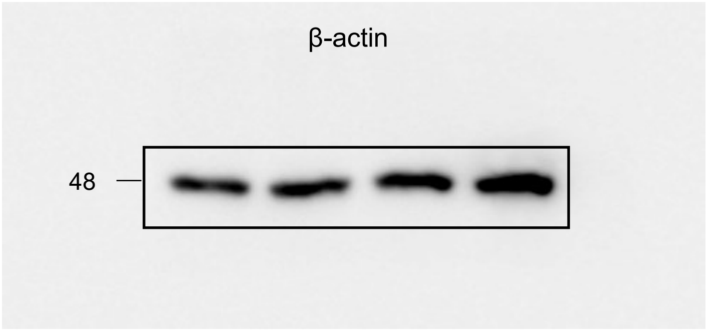

Supplemantary Figure 3 D

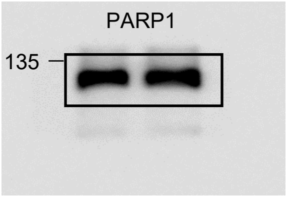

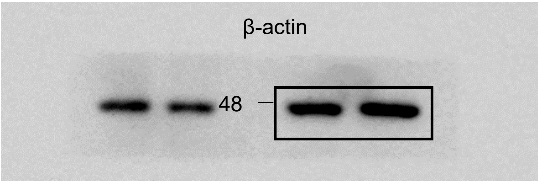

Supplemantary Figure 3 E

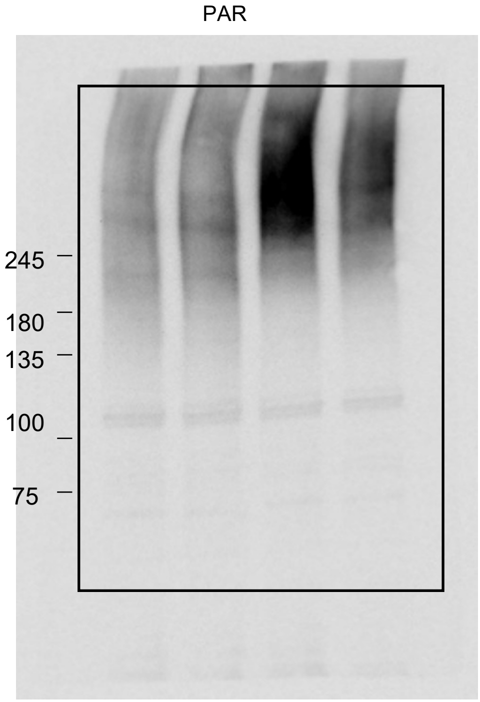

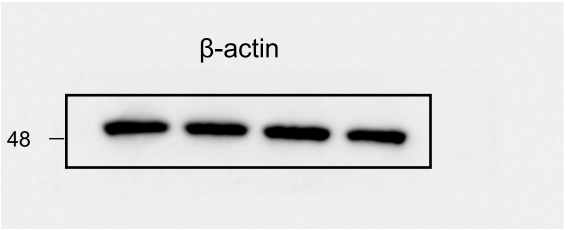

Supplemantary Figure 3 F

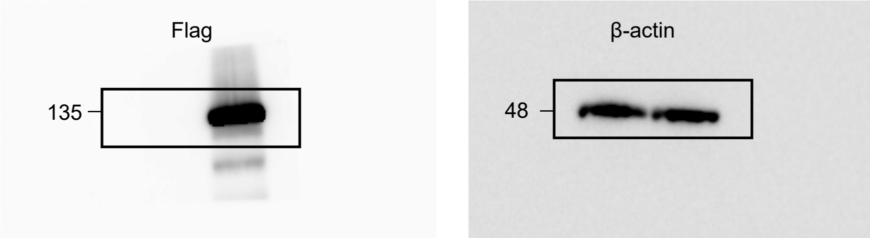

Supplemantary Figure 3 G

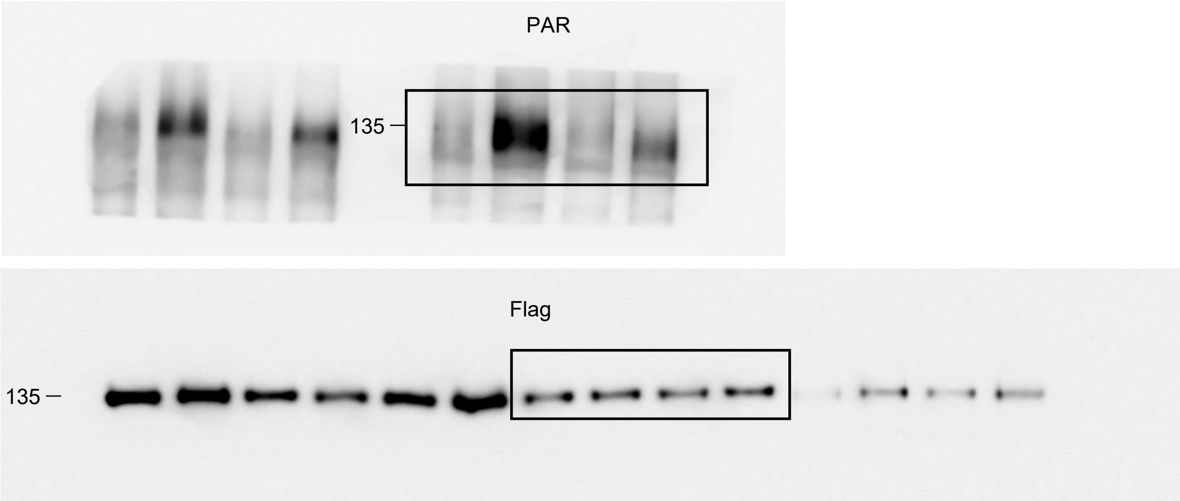

Supplemantary Figure 3 H

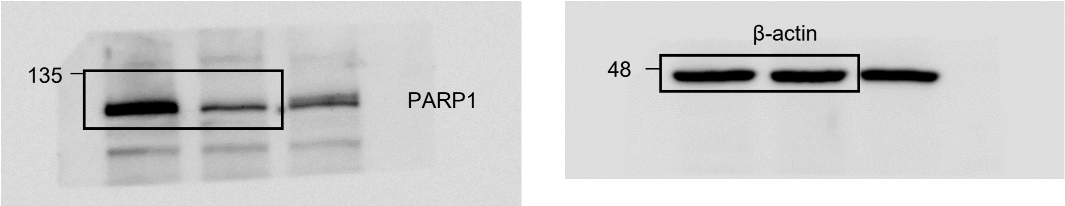

Supplemantary Figure 5 A

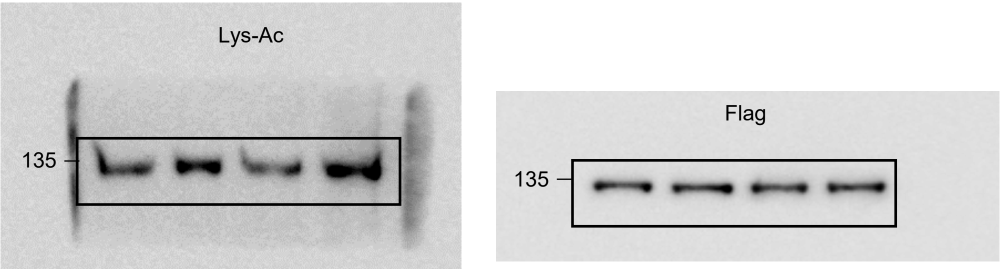

Supplemantary Figure 5 B

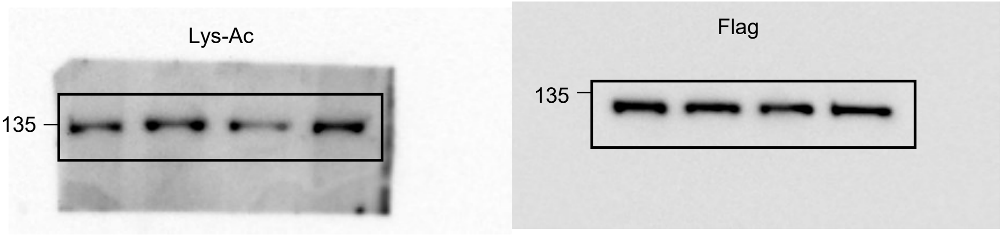

Supplemantary Figure 5 C

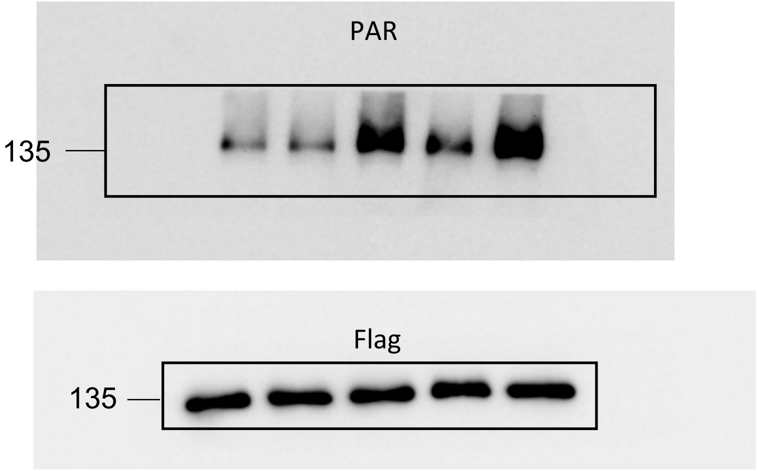

Supplemantary Figure 5 E

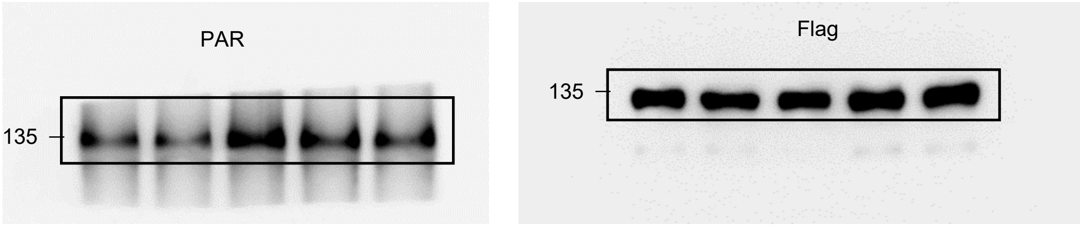

Supplement: Supplementary file 4 — full and uncropped western blots [file 41419_2023_5968_MOESM4_ESM.pdf]
