# Supplementary material for: Fatty acid oxidation facilitates DNA double-strand break repair by promoting PARP1 acetylation
Source: Cell Death Dis. 2023 Jul 15;14(7):435. doi: 10.1038/s41419-023-05968-w (PMC10349888; doi:10.1038/s41419-023-05968-w)
Supplement: Supplementary file 5 — Supplementary Figures [file 41419_2023_5968_MOESM5_ESM.pdf]

Supplementary Figure 1

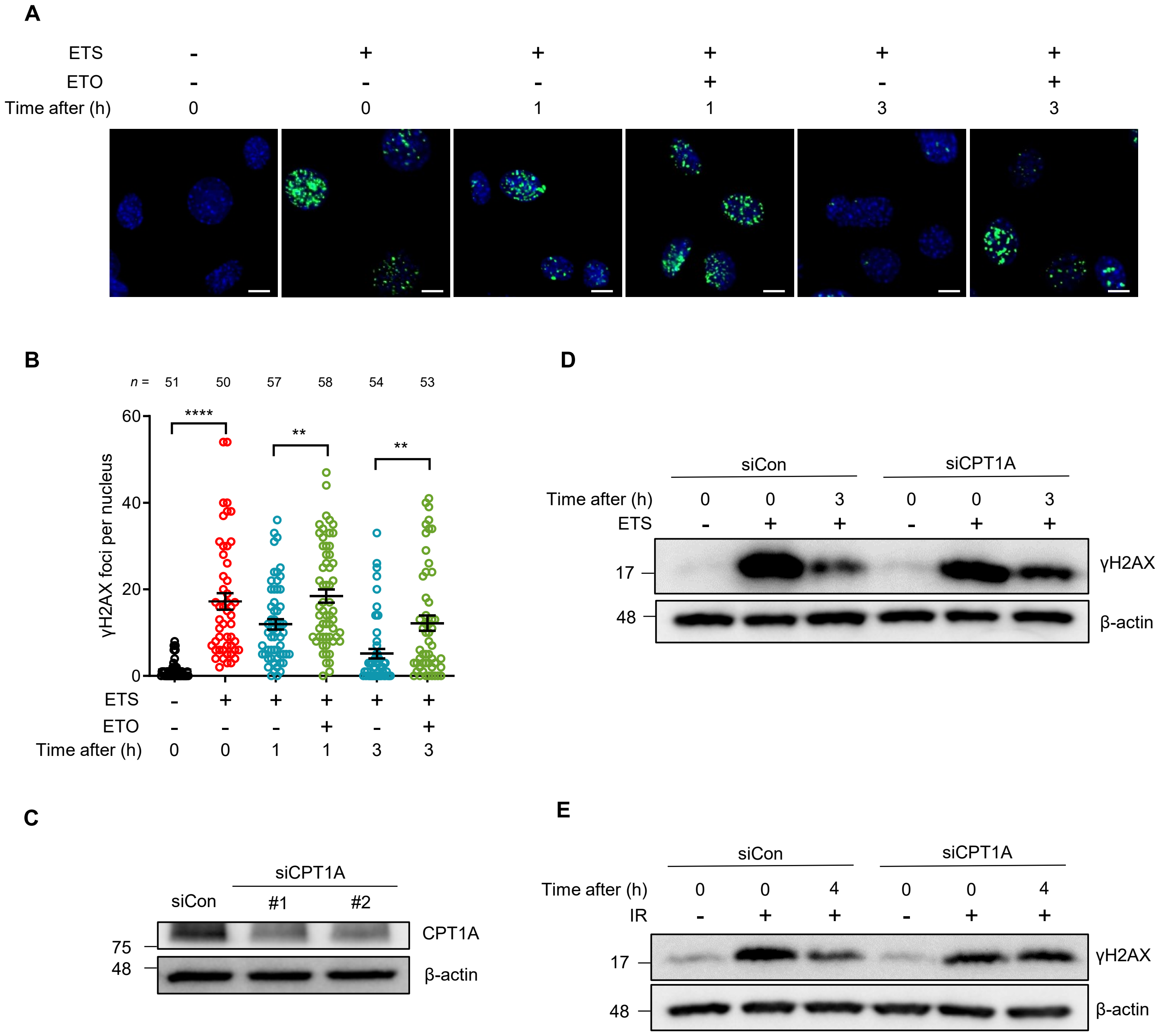

Supplementary Figure 1. FAO is required for DNA double-strand break repair.

(A) Cells were treated with 10 μM ETS for 30 min and then replaced with fresh culture media with or without ETO for the indicated times. Immunofluorescent staining was performed with a nuclear marker (DAPI) and anti-γH2AX antibody on cells. Scale bar represents 6 μm. (B) Number of γH2AX foci per nucleus in each group as shown in B. Statistical analysis was performed using one-way ANOVA with Tukey's multiple comparisons test. The number of cells pooled from three independent experiments is indicated. (C) Levels of CPT1A in cells expressing a control siRNA or siRNAs to CPT1A. β-actin was used as loading control. (D-E) Immortalized MEFs were transfected with siControl or with siRNAs against CPT1A. After transfection, cells were treated with (D) ETS or (E) 3 Gy of IR. Lysates were immunoblotted with anti-γH2AX antibody. All experiments were performed three times. All error bars ± SEM. **p< 0.01, and ****p< 0.0001.

Supplementary Figure 2

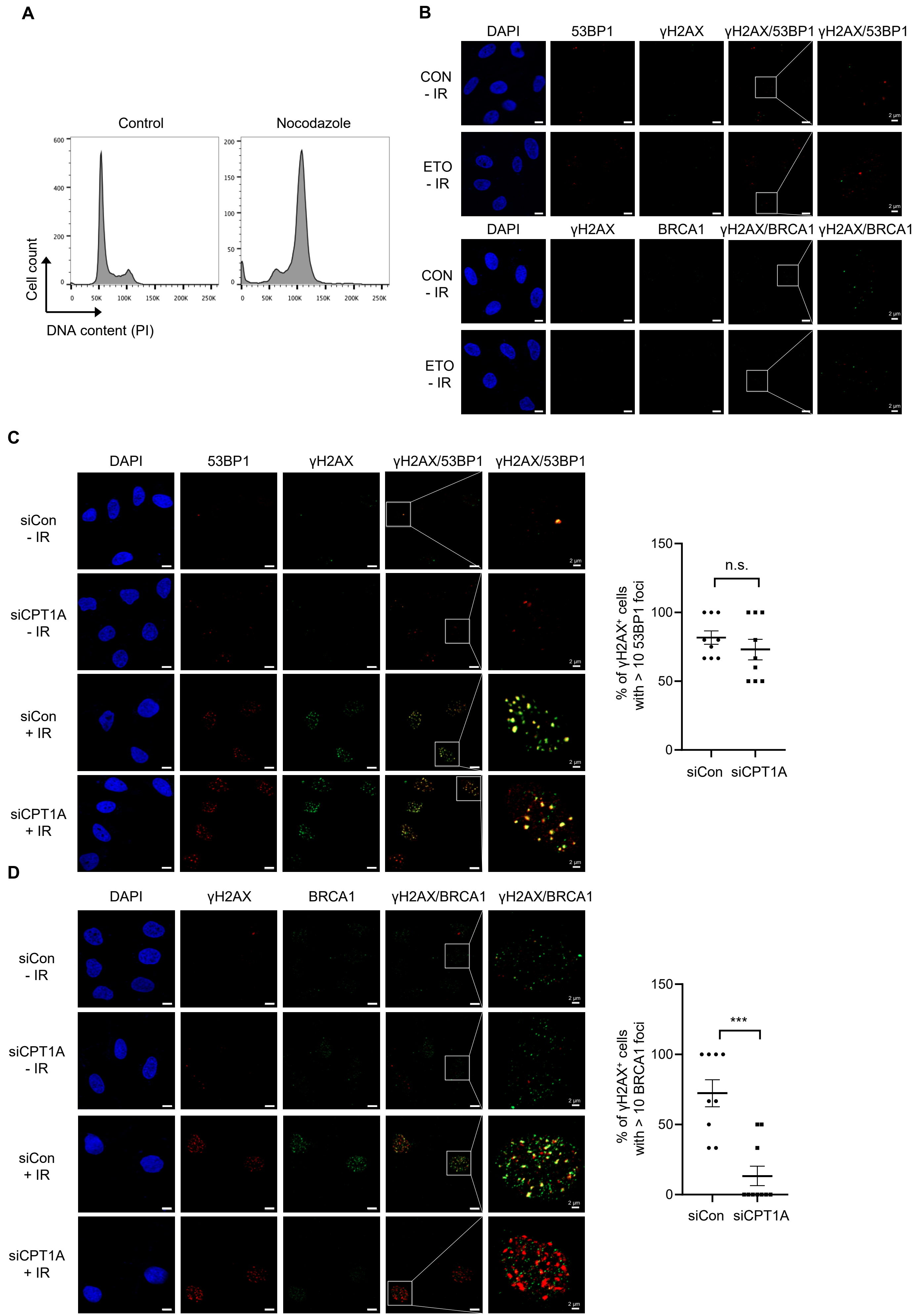

Supplementary Figure 2. FAO promotes the recruitment of BRCA1 to DNA DSBs

(A) Immortalized MEFs were treated with nocodazole (200 nM) for 20 h. Cells were stained with propidium iodide (PI) and analyzed by flow cytometry. (B) Representative images of immunofluorescence staining on the control and ETO treated HeLa cells (53BP1, Red; γH2AX, Green; DAPI, Blue in upper panels and BRCA1, Green; γH2AX, Red; DAPI, Blue in lower panels). Scale bar represents 10 μm. (C-D) Control and CPT1A knockdown HeLa cells were exposed to 3 Gy of IR and harvested 48 h later. Representative images of immunofluorescence staining (53BP1, Red; γH2AX, Green; DAPI, Blue in (C) and BRCA1, Green; γH2AX, Red; DAPI, Blue in (D)). Scale bar represents 10 μm. Percentages of γH2AX positive cells with > 10 Foci of BRCA1 or 53BP1 as indicated (right). Statistical analysis was based on two-tailed Student's t test. All experiments were performed three times. All error bars ± SEM. ***p< 0.001.

Supplementary Figure 3

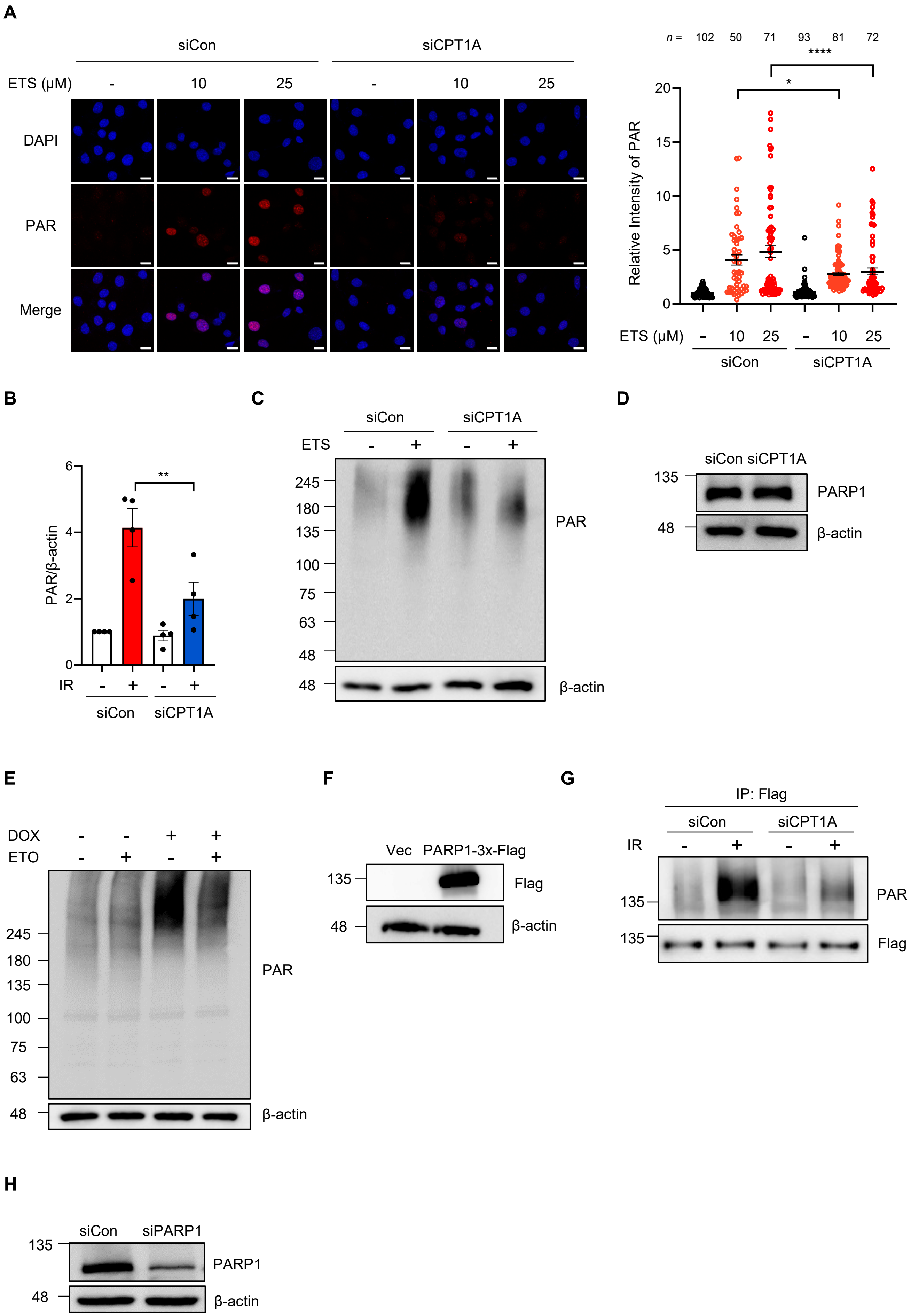

Supplementary Figure 3. FAO supports PARP1 activity upon DNA damage.

(A) Immortalized MEFs were transfected with siControl or siRNAs against CPT1A. Cells were treated with 10 μM PDD00017273 for 1 h and then exposed to 10 μM or 25 μM ETS for 30 min. Immunofluorescent staining was performed with anti-PAR antibody and DAPI. Scale bar represents 20 μm. Relative fluorescence intensity of PAR as indicated (right). Statistical analysis was performed using two-way ANOVA with Tukey's multiple comparisons test. The number of cells pooled from three independent experiments is indicated. (B) 293T cells were transfected with siControl or siRNA against CPT1A and were treated with 10 μM PDD00017273 for 1 h before irradiation (10 Gy). Relative PAR levels as indicated. Statistical analysis was based on two-way ANOVA with Tukey's multiple comparisons test. (C) 293T cells were transfected with siControl or with siRNAs against CPT1A. Cells were pretreated with 10 μM PDD00017273 for 1 h and then exposed to 10 μM ETS overnight. Lysates were immunoblotted with anti-PAR and anti-PARP1 antibodies. (D) PARP1 protein levels in cells transfected with siControl or with siRNA against CPT1A (siCPT1A). β-actin is used as loading control. (E) Immortalized MEFs were treated with ETO and/or 1 μM DOX overnight and incubated PDD00017273 for 1 h. (F) 293T cells were transfected with pCMV-PARP1-3x-Flag for 48 h. Lysates were immunoblotted with Flag antibody. (G) Control or CPT1A knockdown 293T cells were transfected with pCMV-PARP1-3x Flag and treated with 10 μM PDD00017273 for 1 h before irradiation. Lysates were immunoprecipitated with Flag antibody and immunoblotted with anti-PAR antibody. (H) PARP1 protein levels in cells expressing a siControl or siPARP1. All experiments were performed three times. All error bars ± SEM. *$p < 0.05$, **$p < 0.01$ and ****$p < 0.0001$.

Supplementary Figure 4

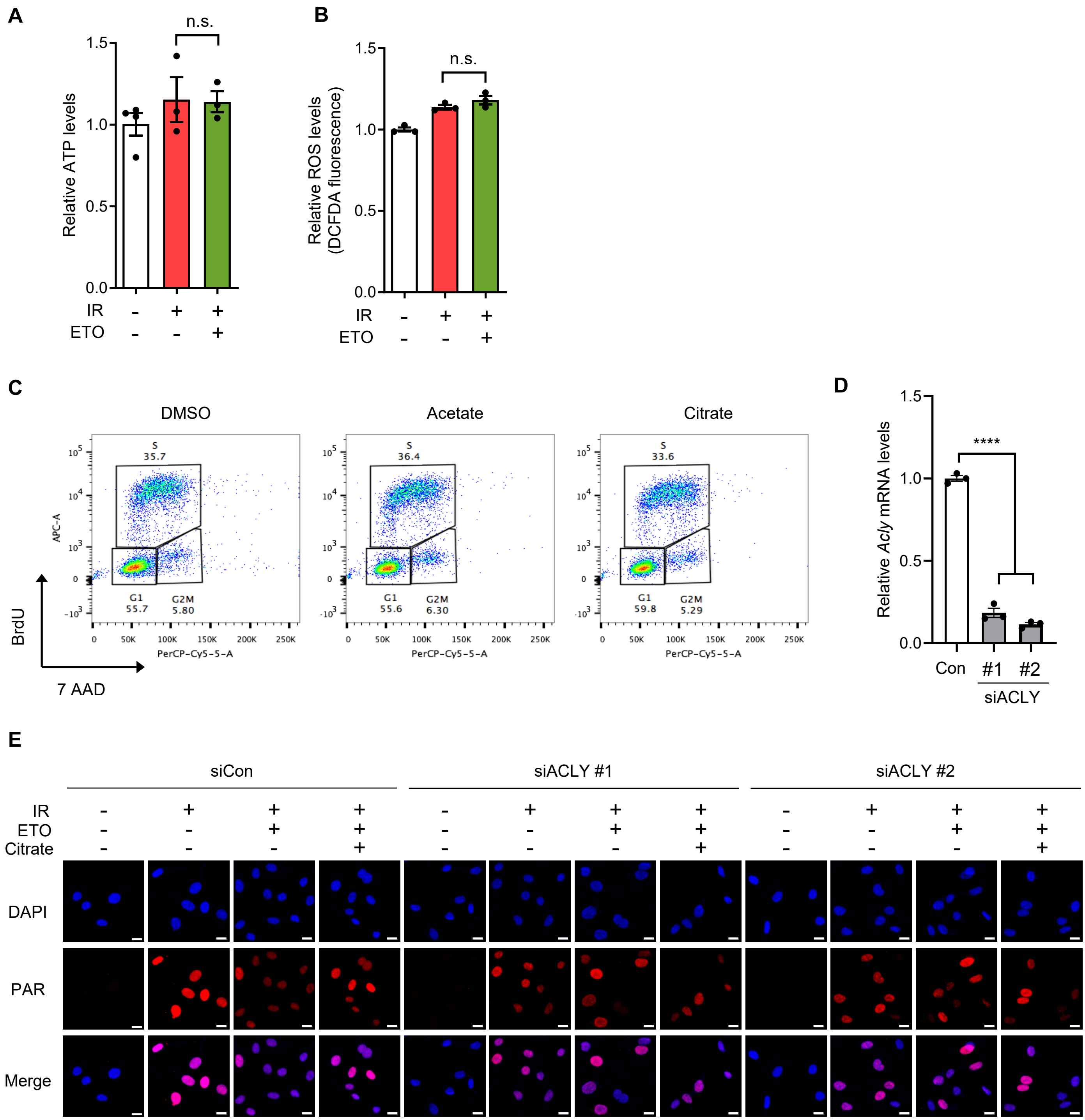

Supplementary Figure 4. FAO contributes to DNA repair through acetyl-CoA

(A and B) Relative (A) ATP or (B) ROS levels of MEFs treated with 3 Gy IR with or without ETO for 4 h. Statistical analysis was performed using one-way ANOVA with Tukey's multiple comparisons test. (C) Immortalized MEFs were treated with 50 mM acetate or 3 mM citrate for 4 h and then incubated with BrdU for 1 h. The cell cycle was analyzed using FACS. (D) Relative *Acly* mRNA levels in MEFs cells transfected with siControl or two independent siRNAs to ACLY (siACLY). Statistical analysis was performed using one-way ANOVA with Tukey's multiple comparisons test. (E) Representative images of immunofluorescence staining in indicated cells. Immortalized MEFs were transfected with siControl or two independent siRNAs to ACLY (siACLY) and treated with the indicated drugs. Immunofluorescent staining was performed with a nuclear marker (DAPI) and anti-PAR antibody on cells. Scale bar represents 20 μm. All experiments were performed three times. All error bars ± SEM. ****$p < 0.0001$.

Supplementary Figure 5

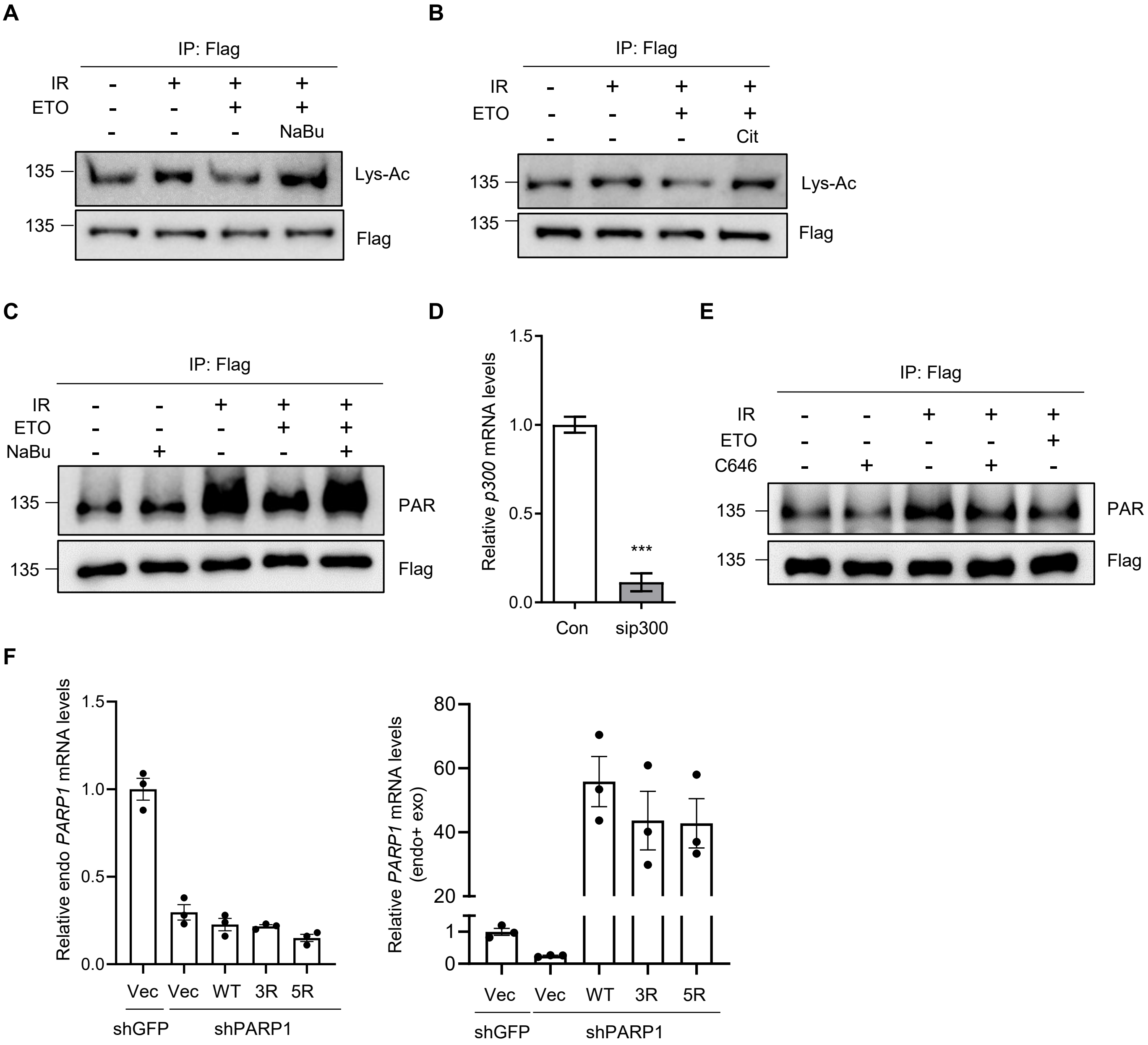

Supplementary Figure 5. Acetylation of PARP1 regulates its activity.

(A-B) 293T cells were transfected with pCMV-PARP1-3x Flag. (A) Transfected cells were irradiated 10 Gy IR and treated with or without ETO and/or (A) 2 mM sodium butyrate (NaBu), and (B) 3 mM citrate for 4 h. Lysates were immunoprecipitated with Flag antibody and immunoblotted with anti-Lys-Ac antibody. (C) 293T cells were transfected with pCMV-PARP1-3x Flag, treated with or without 2 mM sodium butyrate (NaBu) for 4 h and then treat 10 μM PDD00017273 for 1 h before irradiation. Lysates were immunoprecipitated with Flag antibody and immunoblotted with anti-PAR antibody. (D) Relative *p300* mRNA levels in 293T cells transfected with siControl or with siRNA against p300. Statistical analysis was based on two-tailed Student's t-test. (E) 293T cells were transfected with pCMV-PARP1-3x Flag. Transfected cells were treated with ETO or 10 μM C646 for 4 h and then treated with 10 μM PDD00017273 for 1 h before irradiation (10 Gy IR). Lysates were immunoprecipitated with Flag antibody and immunoblotted with anti-PAR antibody. (F) Relative *PARP1* mRNA levels in the indicated cells. 293T cells were infected shGFP or shPARP1 and selected by puromycine (2 μg/ml). After selection, cells were transfected with mock vector, PARP1 wild type (WT), or two mutant forms of PARP1. Expression of endogenous *PARP1* mRNA was determined using PARP1 3' UTR detecting primers (left) and total *PARP1* mRNA expression was analysed using PARP1 CDS detecting primers (right). All experiments were performed three times. All error bars ± SEM. ***p< 0.001.

Supplementary Figure 6

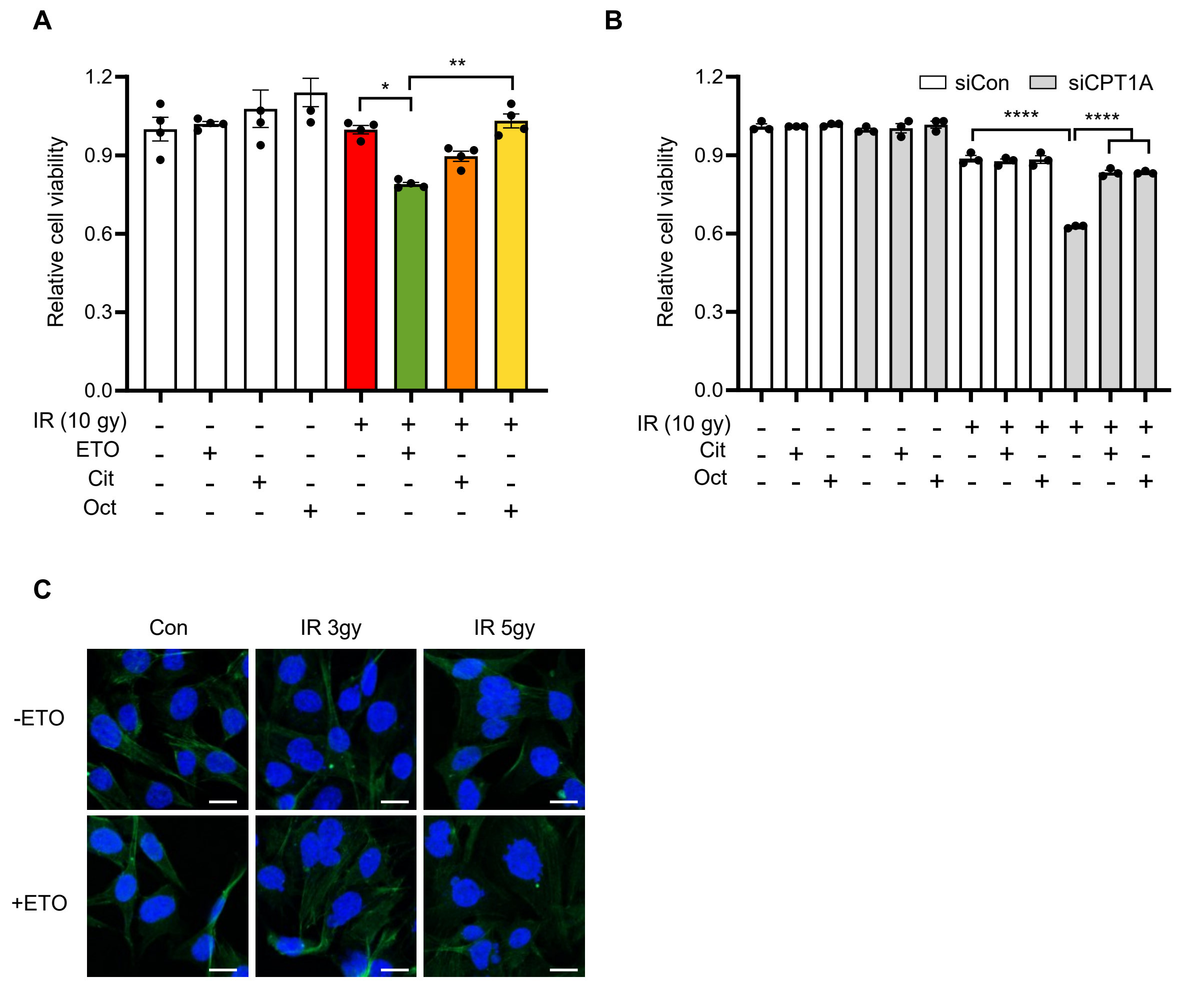

D

	Con (n=405)	ETO (n=408)	IR 3gy (n=399)	IR 3gy+ETO (n=400)	IR 5gy (n=478)	IR 5gy+ETO (n=316)
**Mononucleated**	399 (98.52%)	403 (98.77%)	371 (92.98%)	350 (87.50%)	411 (85.98%)	253 (80.06%)
**Binucleated**	6 (1.48%)	5 (1.23%)	16 (4.01%)	27 (6.75%)	34 (7.11%)	28 (8.86%)
**Multinucleated**	0 (0%)	0 (0%)	12 (3.01%)	23 (5.75%)	33 (6.90%)	35 (11.08%)

Supplementary Figure 6. FAO supports genomic integrity.

(A-B) Cell viability was analyzed using CCK-8 assay. (A) Immortalized MEFs were exposed to 10 Gy of IR and then treated with or without 200 μM ETO, 0.5 mM citrate, and/or 50 μM octanoate for 48 h. Statistical analysis was based on one-way ANOVA with Tukey's multiple comparisons test. (B) Immortalized MEFs were transfected with siControl or siRNAs to CPT1A. Cells were exposed to 10 Gy of IR and then treated with or without 0.5 mM citrate and/or 50 μM octanoate for 48 h. Statistical analysis was performed using two-way ANOVA with Tukey's multiple comparisons test. (C-D) Immortalized MEFs were exposed to 3 Gy or 5 Gy IR and then treated with or without ETO for 48 h. (C) Immunofluorescent staining was performed with anti-phalloidin antibody and DAPI. Scale bar represents 20 μm. (D) Percentages of multinucleated, binucleated, and mononucleated cells as shown in (C). The number of cells pooled from three independent experiments is indicated.
